# The *Toxoplasma gondii* Rhoptry Kinome Is Essential for Chronic Infection

**DOI:** 10.1128/mBio.00193-16

**Published:** 2016-05-10

**Authors:** Barbara A. Fox, Leah M. Rommereim, Rebekah B. Guevara, Alejandra Falla, Miryam Andrea Hortua Triana, Yanbo Sun, David J. Bzik

**Affiliations:** Department of Microbiology and Immunology, Geisel School of Medicine at Dartmouth, Lebanon, New Hampshire, USA

## Abstract

Ingestion of the obligate intracellular protozoan parasite *Toxoplasma gondii* causes an acute infection that leads to chronic infection of the host. To facilitate the acute phase of the infection, *T. gondii* manipulates the host response by secreting rhoptry organelle proteins (ROPs) into host cells during its invasion. A few key ROP proteins with signatures of kinases or pseudokinases (ROPKs) act as virulence factors that enhance parasite survival against host gamma interferon-stimulated innate immunity. However, the roles of these and other ROPK proteins in establishing chronic infection have not been tested. Here, we deleted 26 *ROPK* gene loci encoding 31 unique ROPK proteins of type II *T. gondii* and show that numerous ROPK proteins influence the development of chronic infection. Cyst burdens were increased in the Δ*rop16* knockout strain or moderately reduced in 11 *ROPK* knockout strains. In contrast, deletion of *ROP5*, *ROP17*, *ROP18*, *ROP35*, or *ROP38*/*29*/*19* (*ROP38*, *ROP29*, and *ROP19*) severely reduced cyst burdens. Δ*rop5* and Δ*rop18* knockout strains were less resistant to host immunity-related GTPases (IRGs) and exhibited >100-fold-reduced virulence. ROP18 kinase activity and association with the parasitophorous vacuole membrane were necessary for resistance to host IRGs. The Δ*rop17* strain exhibited a >12-fold defect in virulence; however, virulence was not affected in the Δ*rop35* or Δ*rop38*/*29*/*19* strain. Resistance to host IRGs was not affected in the Δ*rop17*, Δ*rop35*, or Δ*rop38*/*29*/*19* strain. Collectively, these findings provide the first definitive evidence that the type II *T. gondii* ROPK proteome functions as virulence factors and facilitates additional mechanisms of host manipulation that are essential for chronic infection and transmission of *T. gondii*.

## IMPORTANCE

Reactivation of chronic *Toxoplasma gondii* infection in individuals with weakened immune systems causes severe toxoplasmosis. Existing treatments for toxoplasmosis are complicated by adverse reactions to chemotherapy. Understanding key parasite molecules required for chronic infection provides new insights into potential mechanisms that can interrupt parasite survival or persistence in the host. This study reveals that key secreted rhoptry molecules are used by the parasite to establish chronic infection of the host. Certain rhoptry proteins were found to be critical virulence factors that resist innate immunity, while other rhoptry proteins were found to influence chronic infection without affecting virulence. This study reveals that rhoptry proteins utilize multiple mechanisms of host manipulation to establish chronic infection of the host. Targeted disruption of parasite rhoptry proteins involved in these biological processes opens new avenues to interfere with chronic infection with the goal to either eliminate chronic infection or to prevent recrudescent infections.

## INTRODUCTION

*Toxoplasma gondii* chronically infects many warm-blooded vertebrates. Infection with *T. gondii* begins after oral ingestion of tissue cysts or sporocysts. The ensuing acute infection is characterized by rapidly replicating tachyzoite stage parasites that disseminate widely into host tissues (1) until the infection is controlled by host T cell responses and gamma interferon (IFN-γ) (2). While most human infections go unnoticed in immunocompetent individuals, a primary infection during pregnancy can spread through the transplacental route to the fetus, resulting in fetal death or significant congenital disease (1). Prior to elimination of the acute stage infection, tachyzoites access vascular endothelial cells as a replicative niche to breach the blood-brain barrier and enter the central nervous system (3). The replicating tachyzoite stage differentiates into a chronic bradyzoite stage encased in cyst structures that persist to establish chronic infection of the host (4). Neurons are the primary target cell type in which cysts develop in the brain (5, 6). Reactivation of chronic *T. gondii* cysts when host immunity wanes due to HIV/AIDS, chemotherapy, or transplantation therapy causes toxoplasmosis, a severe opportunistic infection of humans (7). Though bradyzoite stage differentiation and the development and maintenance of cyst stages are biologically crucial events for successful transmission of *T. gondii* infection, the biology controlling these stages is not yet well understood. While secreted parasite molecules have been shown to enhance parasite survival by manipulation of host cells and innate immunity during acute infection (8, 9), few secreted parasite molecules have been identified as playing crucial roles in successful chronic infection *in vivo* (10–12).

Comparative genome analysis reveals amplification and diversification of secretory pathogenesis determinants, including rhoptry organelle proteins (ROPs), are key features that distinguish genomes of biologically diverse coccidian parasites (13). ROP molecules play important roles in invasion, in establishing the replicative niche of the parasitophorous vacuole, in host cell manipulation, and in resisting host IFN-γ-stimulated innate immunity (8, 9, 14). Genetic investigations of *T. gondii* strain types that differ in virulence in mice identified ROP5 (15–18) and ROP18 (15, 18–21) as key virulence factors.

ROPs are secreted from *T. gondii* rhoptry organelles directly into host cells during host cell invasion. After their secretion into host cells, ROP5 and ROP18 tether to the cytosolic face of parasitophorous vacuole (PV) membrane (PVM) through N-terminal arginine-rich amphipathic helix (RAH) domains (19, 22–24). PVM-associated ROP18 phosphorylates host immunity-related GTPases (IRGs) to prevent their accumulation on the PVM and thereby preserve the integrity of the PV from destruction (19, 25). Association of the ROP5 pseudokinase with ROP18 increases ROP18 kinase activity (26), and most likely the availability of host IRG substrates for their subsequent inactivation by the ROP18 kinase (27–29). ROP5 also associates in macromolecular complexes with ROP17, another PVM-associated kinase that phosphorylates and inactivates IRGs (22).

In laboratory strains of mice, South American and type I strains are virulent, type II strains exhibit reduced virulence, and type III strains are avirulent (30). While type I PVs avoid IRG accumulation (19), type II and type III PVs accumulate IRGs and are efficiently destroyed (31, 32). To explain these virulence differences in archetypal clonal lineages, virulent ROP18 gene alleles were proposed to inhibit accumulation of IRG proteins on the PVs of strain types that also express a virulent *ROP5* locus (27, 28). This hypothesis explains increased virulence in type I strains that possess virulent *ROP18* and *ROP5* gene alleles. This hypothesis also explains avirulent type III strains that possess a virulent *ROP5* locus and an avirulent and nonexpressed *ROP18* gene allele. However, it is currently unclear how less virulent type II strains that carry a virulent *ROP18* gene allele and an avirulent *ROP5* gene locus resist host IFN-γ to establish chronic infection.

In addition to ROP5, ROP17, and ROP18, many ROP proteins are active kinases or pseudokinases (ROPKs), and the ROPKs collectively are characterized as the rhoptry kinome. The *T. gondii* rhoptry kinome was originally identified to consist of a set of 34 unique ROPK proteins and 10 additional degenerate *ROPK* genes that contain large DNA insertions or deletions within their kinase domains (33). Subsequently, four additional *T. gondii* ROPK genes were identified as *ROP47*, *ROP48*, *ROP49*, and *ROP50* (34). Targeted deletion of *ROP47* or *ROP48* in a type II strain does not affect cyst burdens or chronic infection *in vivo* (35). However, the roles of most ROPKs in virulence, cyst development, and chronic infection in less virulent type II strains have not been directly tested. Here, we developed gene deletions of 26 *ROPK* gene loci encoding 31 unique type II ROPK proteins and examined the ability of these mutants to establish chronic infection in mice. Our results show that while several type II ROPK molecules moderately affect cyst burdens, ROP5, ROP17, ROP18, ROP35, and ROP38/29/19 (ROP38, ROP29, and ROP19) are essential for chronic infection.

## RESULTS

### Type II rhoptry kinome molecules are essential for chronic infection.

**FIG 1:**
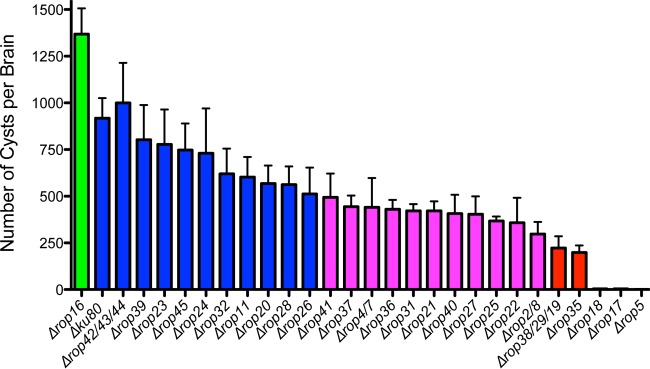
Rhoptry kinome molecules are essential for chronic infection. C57BL/6 mice were infected i.p. with 200 tachyzoites of each strain, and brain cyst burdens were measured 21 days postinfection. Data shown are cumulative results from one to three independent experiments for each strain tested. *ROPK* knockout strains and the Δ*ku80* parent strain are organized in descending order by cyst number for each strain, with cyst numbers shown as means plus standard errors of the means (SEM) (error bars). *P* values were calculated by a Student’s *t* test, and a *P* < 0.05 (shown in purple or red color) was considered significant. Data on the ROPK knockout strains and the Δ*ku80* parent strain follow: Δ*rop16* (two experiments; *n* = 6 mice; *P* = 0.0242), Δ*ku80* parent (three experiments; *n* = 12 mice), Δ*rop42*/*43*/*44* (one experiment; *n* = 4 mice; *P* = 0.7188), Δ*rop39* (two experiments; *n* = 7 mice; *P* = 0.5691), Δ*rop23* (one experiment; *n* = 4 mice; *P* = 0.5232), Δ*rop45* (one experiment; *n* = 4 mice; *P* = 0.4186), Δ*rop24* (one experiment; *n* = 4 mice; *P* = 0.4259), Δ*rop32* (one experiment; *n* = 4 mice; *P* = 0.1651), Δ*rop11* (two experiments; *n* = 7 mice; *P* = 0.0703), Δ*rop20* (two experiments; *n* = 6 mice; *P* = 0.0534), Δ*rop28* (one experiment; *n* = 4 mice; *P* = 0.0928), Δ*rop26* (two experiments; *n* = 5 mice; *P* = 0.0500), Δ*rop41* (two experiments; *n* = 5 mice; *P* = 0.0384), Δ*rop37* (two experiments; *n* = 5 mice; *P* = 0.0154), Δ*rop4*/*7* (three experiments; *n* = 12 mice; *P* = 0.0198), Δ*rop36* (two experiments; *n* = 6 mice; *P* = 0.0070), Δ*rop31* (two experiments; *n* = 6 mice; *P* = 0.0058), Δ*rop21* (two experiments; *n* = 7 mice; *P* = 0.0036), Δ*rop40* (one experiment; *n* = 4 mice; *P* = 0.0217), Δ*rop27* (two experiments; *n* = 6 mice; *P* = 0.0073), Δ*rop25* (two experiments; *n* = 7 mice; *P* = 0.0013), Δ*rop22* (two experiments; *n* = 5 mice; *P* = 0.0095), Δ*rop2*/*8* (one experiment; *n* = 4 mice; *P* = 0.0063), Δ*rop38*/*29*/*19* (two experiments; *n* = 8 mice; *P* < 0.0001), Δ*rop35* (two experiments; *n* = 8 mice; *P* < 0.0001), Δ*rop18* (two experiments; *n* = 8 mice; *P* < 0.0001), Δ*rop17* (two experiments; *n* = 8 mice; *P* < 0.0001), and Δ*rop5* (three experiments; *n* = 9 mice; *P* < 0.0001).

To broadly assess the roles of type II ROPK proteins in chronic infection, we targeted the deletion of 27 previously defined unique *ROPK* gene loci that encode 32 identified members of the *ROPK* gene family (*ROP2*/*8*, *ROP5*, *ROP11*, *ROP16*, *ROP17*, *ROP18*, *ROP20*, *ROP21*, *ROP22*, *ROP23*, *ROP24*, *ROP25*, *ROP26*, *ROP27*, *ROP28*, *ROP30*, *ROP31*, *ROP32*, *ROP35*, *ROP36*, *ROP37*, *ROP38*/*29*/*19*, *ROP39*, *ROP40*, *ROP41*, *ROP42*/*43*/*44*, and *ROP45*) (33). Additionally, we previously reported the deletion of the type II *ROP4*/*7* locus (11). *ROPK* gene locus deletions were targeted in the Δ*ku80* genetic model in the *Toxoplasma gondii* type II Prugniaud (Pru) background (11), and knockout genotypes were validated by PCR to measure targeted deletion of the *ROPK* gene locus and the correct integration of the *HXGPRT* selectable marker into the deleted *ROPK* gene locus (see Fig. S1A in the supplemental material) (36, 37). We validated the isolation of 26 *ROPK* gene locus knockouts (see Table S1 in the supplemental material). We assessed the roles of specific *ROPK* proteins in establishing chronic infection *in vivo* by measuring brain cyst burdens in C57BL/6 mice infected with *ROPK* knockout strains (Fig. 1). One *ROPK* knockout (Δ*rop16*) exhibited a significant increase in cyst burdens, and cyst burdens were not significantly modified in 10 other *ROPK* knockout strains (Δ*rop11*, Δ*rop20*, Δ*rop23*, Δ*rop24*, Δ*rop26*, Δ*rop28*, Δ*rop32*, Δ*rop39*, Δ*rop42*/*43*/*44*, and Δ*rop45* strains). In contrast, cyst burdens were moderately decreased by 46 to 68% in 11 other *ROPK* knockout strains (Δ*rop2*/*8*, Δ*rop4*/*7*, Δ*rop21*, Δ*rop22*, Δ*rop25*, Δ*rop27*, Δ*rop31*, Δ*rop36*, Δ*rop37*, Δ*rop40*, and Δ*rop41* strains). Importantly, cyst burdens were severely decreased by 75 to 80% in Δ*rop35* and Δ*rop38*/*29*/*19* strains and by >99.5% in Δ*rop5*, Δ*rop17*, and Δ*rop18* strains compared to the Δ*ku80* parent (Fig. 1).

### *ROPK* knockout strains with the most severe defects in chronic infection stage differentiate *in vitro*.

**FIG 2:**
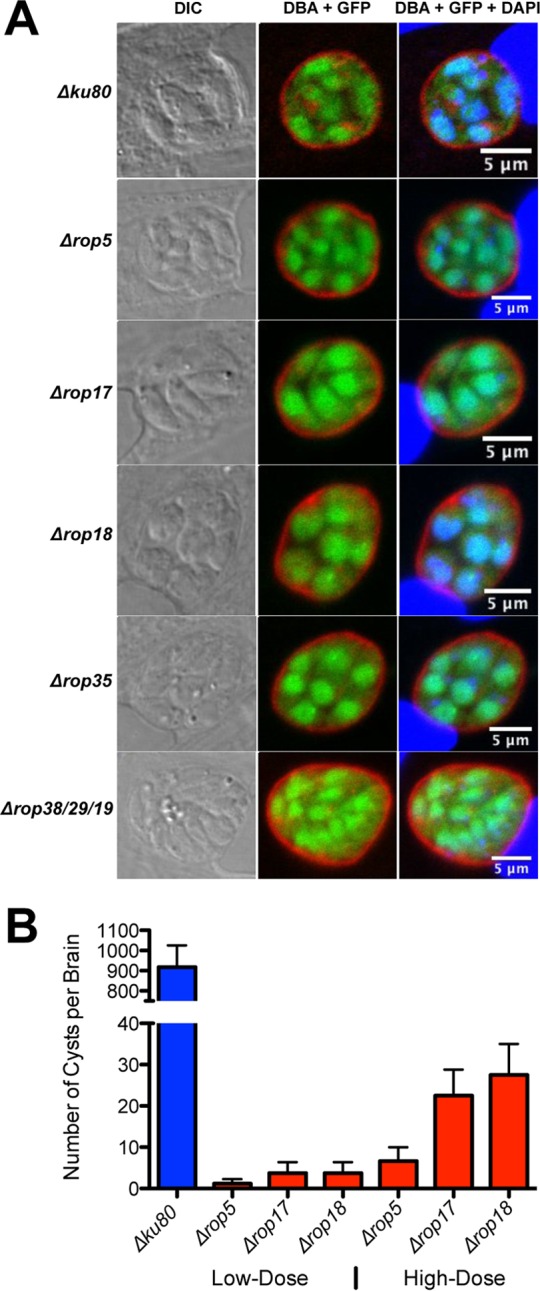
ROPK gene deletions with the most severe defects in cyst burdens differentiate normally *in vitro*. (A) Infected host cells were treated with bradyzoite inducing conditions (pH 8.1 and CO_2_ depletion in ambient air) for 3 days. The cyst wall was stained with *Dolichos biflorus* agglutinin (DBA) (shown in red). Bradyzoites were visualized by expression of GFP (shown in green), which is under control of the bradyzoite stage-specific *LDH2* promoter. Host cell and parasite nuclei were stained with DAPI (shown in blue). Samples were imaged by confocal microscopy, and vacuoles were located using differential interference contrast (DIC) microscopy. Representative results are shown for each strain. (B) C57BL/6 mice were infected i.p. with 200 tachyzoites of each strain (low-dose infection) or with 2 × 10^5^ or 2 × 10^6^ tachyzoites of each strain (high-dose infection), and brain cyst burdens were measured. The values shown represent means plus SEM (error bars). Data shown are cumulative results from one to three independent experiments for each strain tested. Data on the ROPK knockout strains and the Δ*ku80* parent strain follow: Δ*ku80* parent low dose (three experiments; *n* = 12 mice), Δ*rop5* low dose (three experiments; *n* = 9 mice), Δ*rop17* low dose (two experiments; *n* = 8 mice), Δ*rop18* low dose (two experiments; *n* = 8 mice), Δ*rop5* high dose (three experiments; *n* = 6 mice), Δ*rop17* high dose (one experiment; *n* = 4 mice), and Δ*rop18* high dose (two experiments; *n* = 4 mice).

One hypothesis is that *ROPK* knockout strains severely deficient in their ability to establish chronic infection may be defective in differentiation from the tachyzoite stage to the bradyzoite stage (4). To examine this, we used high pH to induce differentiation *in vitro*. Parental Δ*ku80* strain tachyzoites switched normally to the bradyzoite cyst stage based on expression of cytosolic green fluorescent protein (GFP) under control of the bradyzoite stage-specific *LDH2* gene promoter (11) and on the development of the cyst wall structure that surrounds the bradyzoites as assessed by *Dolichos biflorus* agglutinin (DBA) lectin staining of the major cyst wall protein CST1 (12) (Fig. 2A). The Δ*rop5*, Δ*rop17*, Δ*rop18*, Δ*rop35*, and Δ*rop38*/*29*/*19* knockout strains exhibited no defect in their ability to differentiate to cyst stages *in vitro* (Fig. 2A) or in their frequency of differentiation (data not shown). We next examined whether mice infected with high doses (>10^5^ tachyzoites) of Δ*rop5*, Δ*rop17*, or Δ*rop18* tachyzoites could rescue normal cyst burdens. Cyst burdens remained extremely low even after high-dose infection of mice with Δ*rop5*, Δ*rop17*, or Δ*rop18* tachyzoites (Fig. 2B). Therefore, it was not a lack of differentiation that hindered cyst development in these knockout strains.

### Single wild-type gene alleles rescue the cyst defect in Δ*rop17* and Δ*rop18* knockout strains, but not in the Δ*rop5* knockout strain.

**FIG 3:**
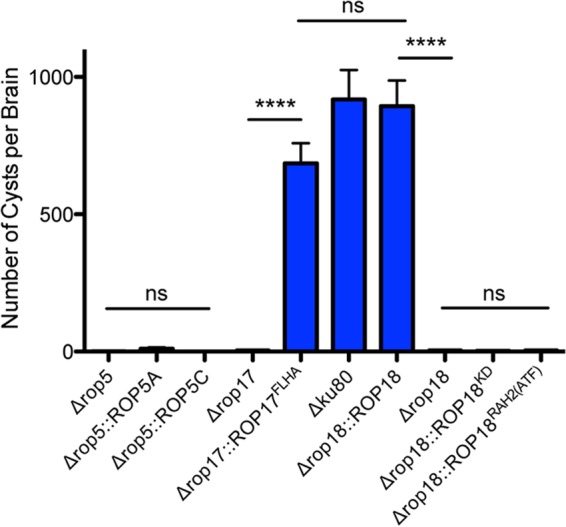
Complementation of Δ*rop17* and Δ*rop18* with the corresponding wild-type gene alleles rescues cyst burdens. C57BL/6 mice were infected i.p. with 200 tachyzoites of each strain, and brain cyst burdens were measured 21 days postinfection. Data shown are cumulative results from one to three independent experiments for each strain tested. Data on the Δ*rop17* and Δ*rop18* knockout strains and the Δ*ku80* parent strain follow: Δ*ku80* parent (three experiments; *n* = 12 mice), Δ*rop5* (three experiments; *n* = 9 mice), Δ*rop5*::*ROP5A* (two experiments; *n* = 8 mice), Δ*rop5*::*ROP5C* (two experiments; *n* = 8 mice), Δ*rop17* (two experiments; *n* = 8 mice), Δ*rop17*::*ROP17^FLHA^* (one experiment; *n* = 4 mice), Δ*rop18* (two experiments; *n* = 8 mice), Δ*rop18*::*ROP18* (two experiments; *n* = 8 mice), Δ*rop18*::*ROP18^KD^* (two experiments; *n* = 8 mice), Δ*rop18*::*ROP18*^*RAH2*(*ATF*)^ (two experiments; *n* = 8 mice). The values shown represent means ± SEM. The *P* values were calculated with a Student’s *t* test. *P* values that were significantly different are indicated by a bar and four asterisks (*P* < 0.0001). *P* values that were not significantly different (ns) are also indicated.

The Δ*rop5*, Δ*rop17*, and Δ*rop18* knockout strains were complemented with their corresponding wild-type gene alleles targeted into the *UPRT* gene locus to simultaneously delete *UPRT* and enable selection of complemented strains in 5-fluorodeoxyuridine (FUDR) (see Fig. S1B in the supplemental material). Complementation of Δ*rop17* with C-terminal FLAG/hemagglutinin (HA)-tagged *ROP17* (*ROP17^FLHA^*) (Fig. S2A) rescued cyst burdens (Fig. 3). The Δ*rop18* knockout strain was complemented with wild-type *ROP18*, a kinase-dead (KD) *ROP18* (*ROP18^KD^*) (19), or a *ROP18* gene allele [*ROP18*^*RAH2*(*ATF*)^] lacking the second RAH domain (RAH2) required for PVM association (23, 24). The ROP18 RAH2 domain also associates with ATF6β (ATF), a host endoplasmic reticulum stress sensor (38). Wild-type *ROP18* fully rescued cyst burdens. However, expression of mutant *ROP18^KD^* or *ROP18*^RAH2(*ATF*)^ gene alleles (Fig. S2B) failed to rescue cyst burdens (Fig. 3).

The type II ME49 strain *ROP5* gene locus is multiallelic and possesses ~10 *ROP5* gene alleles (16, 17). Complementation of Δ*rop5* through expression of the *ROP5A* or *ROP5C* gene allele (see Fig. S2C in the supplemental material) failed to rescue cyst burdens (Fig. 3), even after high-dose infection (data not shown). To examine the *ROP5* locus in the type II Pru strain, we PCR amplified the full coding region of *ROP5A* and *ROP5C* alleles from the Δ*ku80* parent strain and sequenced randomly selected clones. Eight *ROP5C* alleles were observed for each *ROP5A* allele (Fig. S3), suggesting that the Pru strain *ROP5* locus is highly similar to the ME49 strain *ROP5* locus (16, 17).

### The Δ*rop17* knockout strain exhibits mild egress and growth defects *in vitro*.

**FIG 4:**
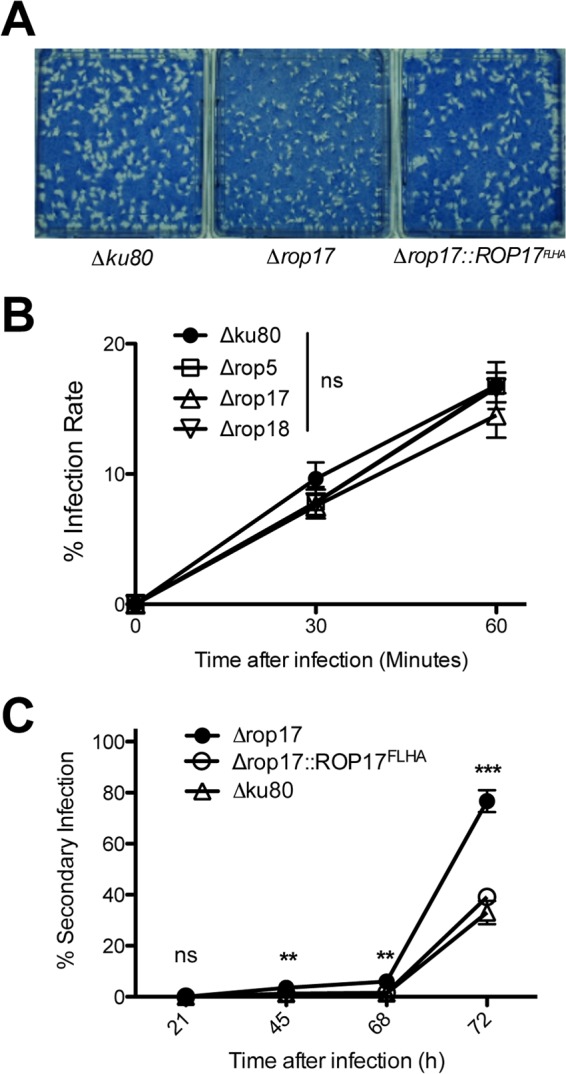
Deletion of ROP17 reduces plaque size and induces early egress. (A) Monolayers of human foreskin fibroblast (HFF) host cells were infected with ~200 tachyzoites of the indicated strains. PFU were visualized 13 days after infection by staining with Coomassie brilliant blue and photographed. The results shown are representative of three independent experiments. (B) Monolayers of HFF cells were infected with the indicated strains, and noninvaded parasites were removed from cultures at 30 or 60 min postinfection and PFU were determined. The infection rate was calculated by comparison to cultures where parasites were not removed (total PFU), and the percent infection rate was determined. Results are from cumulative data obtained in three independent experiments and are shown as means ± SEM (error bars). The values were not significantly different (ns) at 30 min or at 60 min postinfection, calculated with a Student’s *t* test. (C) Monolayers of HFF host cells were infected at an MOI of ~0.01, and noninvaded parasites were removed 1 h postinfection. At 21, 45, 68, or 72 h after infection, cultures were scored microscopically to measure the percentage of secondary infection sites. Results are from cumulative data obtained in three independent experiments and are shown as means ± SEM. The *P* values were calculated with a Student’s *t* test and are indicated as follows: **, *P* < 0.01; ***, *P* < 0.001; ns, not significant.

*ROPK* knockout strains deficient in chronic infection may be compromised in their ability to replicate. To globally assess *ROPK* knockout strains for growth defects, we first examined plaques representing zones of infection where repeated cycles of invasion, intracellular replication, and egress occur. While all other *ROPK* knockout strains and their complemented strains developed plaques that were similar in size to those of the Δ*ku80* parent (data not shown), the Δ*rop17* knockout strain developed smaller plaques (Fig. 4A). This small-plaque phenotype was rescued by expression of the *ROP17^FLHA^* gene (Fig. 4A). However, the Δ*rop17* small-plaque phenotype was not associated with any significant defect in the replication rate (see Fig. S4A in the supplemental material) or in the number of parasites per PV at 21 h (data not shown) or 45 h postinfection (Fig. S4B). In addition, the Δ*rop17*, Δ*rop5*, and Δ*rop18* knockout strains infected host cells as efficiently as the Δ*ku80* parent (Fig. 4B). Consequently, we measured whether the Δ*rop17* strain exhibited any defect in the egress of replicated parasites from the PV. Tachyzoites egressed from a higher percentage of Δ*rop17* PVs after 45, 68, and 72 h of infection, and this early egress phenotype was rescued by complementation with *ROP17^FLHA^* (Fig. 4C). However, the average numbers of Δ*rop17* parasites at secondary sites of infection after release from the PVs were not different from those of the Δ*ku80* parent or the *ROP17^FLHA^* complemented strain (Fig. S4C), suggesting that this early egress phenotype did not immediately alter invasion or continued replication of Δ*rop17* parasites.

### Type II ROP5 and ROP18 molecules actively resist PV killing, but ROP17 does not.

**FIG 5:**
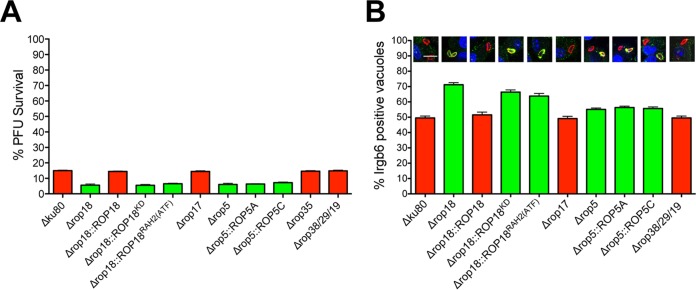
Type II ROP5 and ROP18 resist Irgb6 coating and killing of the PV. (A) MEFs were stimulated with IFN-γ, and parasite survival (measured as PFU) was determined in comparison to nonstimulated MEFs. Results are from at least four independent experiments and are shown as means plus SEM. Significant *P* values were calculated with a Student’s *t* test. The values for all strains shown in red were not significantly different from the value for the Δ*ku80* parent. The values for all strains shown in green were significantly different (*P* < 0.0001) from the value for the Δ*ku80* parent strain. (B) Quantification of Irgb6 coating of PVs 45 min after infection of IFN-γ-stimulated bone marrow-derived macrophages. Representative images are shown of PVs stained with anti-Irgb6 (shown in green) and anti-GRA5 (shown in red). At least 500 PVs were scored to determine significance. Significant *P* values were calculated with a Student’s *t* test. The values for all strains shown in red were not significantly different from the value for the Δ*ku80* parent. The values for all strains shown in green were significantly different from the value for the Δ*ku80* parent strain. The *P* values for the knockout strains shown in green are listed in parentheses after the genotype: Δ*rop18* (*P* < 0.0001), Δ*rop18*::*ROP18^KD^* (*P* < 0.0001), Δ*rop18*::*ROP18*^RAH2(*ATF*)^ (*P* < 0.0001), Δ*rop5* (*P* < 0.0013), Δ*rop5*::*ROP5A* (*P* < 0.0001), and Δ*rop5*::*ROP5C* (*P* < 0.0006).

While virulent type I strains effectively resist host IRGs (19), the PVs of type II strains are highly susceptible to coating and killing by host IRGs (31, 32). As expected, only a low percentage of type II Δ*ku80* PVs (~15%) survived in IFN-γ-stimulated mouse embryonic fibroblasts (MEFs) (Fig. 5A). In contrast, survival of the Δ*rop5* and Δ*rop18* knockout strains was further reduced by ~10% compared to that of the Δ*ku80* parent. Complementation of Δ*rop18* with wild-type *ROP18* rescued resistance to PV killing (Fig. 5A). Moreover, complementation of Δ*rop18* with *ROP18* gene alleles defective in kinase activity (*ROP18^KD^*) or PVM association [*ROP18*^*RAH2*(*ATF*)^], as expected, failed to rescue Δ*rop18* resistance to PV killing. Single-copy gene alleles of *ROP5A* or *ROP5C* also failed to rescue Δ*rop5* resistance to PV killing (Fig. 5A). Single-copy gene alleles of virulent type I *ROP5A* or *ROP5C* were previously reported to incompletely rescue resistance to IRGs or virulence (16, 17, 28). Surprisingly, the Δ*rop17*, Δ*rop35*, and Δ*rop38*/*29*/*19* knockouts exhibited no defect in their resistance to PV killing in IFN-γ-stimulated MEFs (Fig. 5A). 

Virulent type I alleles of ROP5, ROP17, and ROP18 are associated with reduced PV coating by Irgb6 in IFN-γ-stimulated macrophages (22). Irgb6 coating was significantly increased on Δ*rop5* PVs (~55% coating), and a single copy of the *ROP5A* or *ROP5C* gene allele failed to rescue resistance to coating (Fig. 5B). Irgb6 coating on Δ*rop18* PVs was markedly increased (~72% coating), and while wild-type *ROP18* rescued this phenotype, the mutant *ROP18^KD^* or *ROP18*^*RAH2*(*ATF*)^ gene allele failed to rescue resistance to Irgb6 coating (Fig. 5B). In contrast to Δ*rop5* and Δ*rop18* PVs, Δ*rop38*/*29*/*19* and Δ*rop17* PVs exhibited the same resistance to Irgb6 coating as the Δ*ku80* parental strain (Fig. 5B). Collectively, these results suggest that while type II ROP5 and ROP18 resist IRG coating and PV killing, ROP17, ROP35, and ROP39/29/19, individually, were not required for IRG resistance.

### Type II parasites deficient in ROP5 and ROP18 are less virulent than parasites deficient in ROP17, ROP35, or ROP38/29/19.

**FIG 6:**
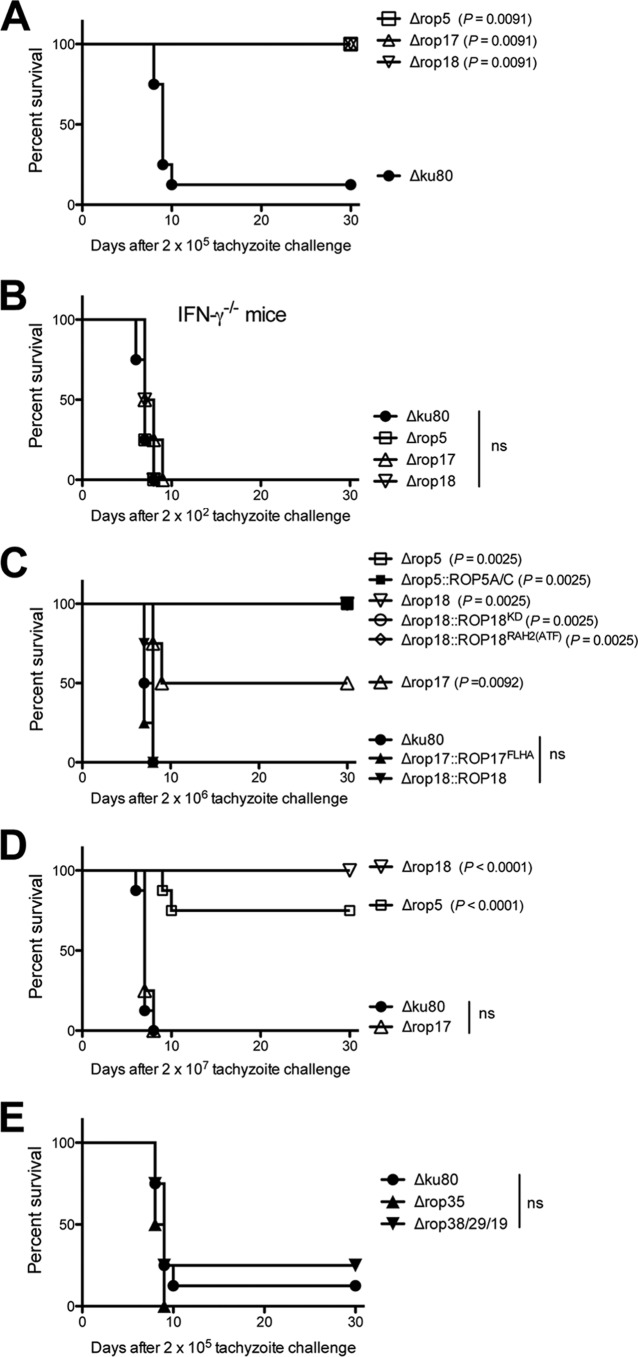
Type II parasites deficient in ROP5 and ROP18 are less virulent than parasites deficient in ROP17, ROP35, or ROP38/29/19. The Δ*rop5*, Δ*rop17*, Δ*rop18*, Δ*rop35*, and Δ*rop38*/*29*/*19* mutants and complemented strains were evaluated for virulence lethality in mice. (A) C57BL/6 mice were infected i.p. with 2 × 10^5^ tachyzoites of the Δ*rop5*, Δ*rop17*, or Δ*rop18* knockout strain or the Δ*ku80* parent strain. Data shown are combined from two independent experiments, each with four mice per group. The *P* values were calculated by a log rank Mantel-Cox test, and a *P* of <0.05 was considered significant. (B) C57BL/6 IFN-γ^−/−^ knockout mice were infected i.p. with 2 × 10^2^ tachyzoites of the Δ*rop5*, Δ*rop17*, or Δ*rop18* strain or the Δ*ku80* parental strain. Data shown are from a single experiment with four mice per group. The *P* values were calculated by a log rank Mantel-Cox test, and a *P* of <0.05 was considered significant. ns, not significant. (C) C57BL/6 mice were infected i.p. with 2 × 10^6^ tachyzoites of the Δ*rop5*, Δ*rop17*, or Δ*rop18* strain or the Δ*ku80* parental strain or with 2 × 10^6^ tachyzoites of the Δ*rop5*::*ROP5A* or Δ*rop5*::*ROP5C* [shown together as Δ*rop5*::*ROP5A*/*C*], Δ*rop17*::*ROP17^FLHA^*, Δ*rop18*::*ROP18*, Δ*rop18*::*ROP18^KD^*, or Δ*rop18*::*ROP18*^*RAH2*(*ATF*)^ complemented strain. Data shown are combined from two independent experiments, each with four mice per group. The *P* values were calculated by a log rank Mantel-Cox test, and a *P* of <0.05 was considered significant. (D) C57BL/6 mice were infected i.p. with 2 × 10^7^ tachyzoites of the Δ*rop5*, Δ*rop17*, or Δ*rop18* strain or the Δ*ku80* parental strain. Data shown are combined from two independent experiments, each with four mice per group. The *P* values were calculated by a log rank Mantel-Cox test, and a *P* of <0.05 was considered significant. (E) C57BL/6 mice were infected i.p. with 2 × 10^5^ tachyzoites of the Δ*rop35* or Δ*rop38*/*29*/*19* strain or the Δ*ku80* parent strain. Data shown are combined from two independent experiments, each with four mice per group.

Virulence of the Δ*rop5*, Δ*rop17*, and Δ*rop18* knockout strains was measured in C57BL/6 mice. The Δ*ku80* parent showed dose-dependent survival, and the 50% lethal dose (LD_50_) was ~1 × 10^5^ tachyzoites (Fig. 6A). In contrast, the Δ*rop5*, Δ*rop17*, and Δ*rop18* knockout strains failed to exhibit virulence lethality after inoculation of mice with 2 × 10^5^ tachyzoites (Fig. 6A). Furthermore, C57BL/6 mice deficient in the production of IFN-γ (IFN-γ^−/−^ mice) failed to control a low-dose infection of Δ*rop5*, Δ*rop17*, or Δ*rop18* tachyzoites (Fig. 6B), suggesting that IFN-γ-stimulated mechanisms were necessary to control infection by these mutants. The Δ*rop17* knockout strain exhibited incomplete virulence lethality after inoculation of 2 × 10^6^ tachyzoites (Fig. 6C). In contrast, all mice survived a 2 × 10^6^ challenge dose of Δ*rop5* or Δ*rop18* tachyzoites (Fig. 6C). Mice infected with 2 × 10^7^ Δ*rop18* tachyzoites also survived infection (Fig. 6D). However, a small fraction of mice infected with 2 × 10^7^ Δ*rop5* tachyzoites succumbed to infection, whereas all mice infected with 2 × 10^7^ Δ*rop17* tachyzoites succumbed (Fig. 6D). The Δ*rop17* LD_50_ was increased ~12-fold compared to that of the Δ*ku80* parent, and the LD_50_ of the Δ*rop5* or Δ*rop18* strain was increased >100-fold. While single *ROP5A* and *ROP5C* gene alleles failed to rescue virulence of the Δ*rop5* knockout strain, the *ROP17^FLHA^* gene allele rescued virulence of the Δ*rop17* knockout strain, and wild-type *ROP18* rescued virulence of the Δ*rop18* knockout strain (Fig. 6C). The mutant *ROP18^KD^* or *ROP18*^RAH2(*ATF*)^ gene allele failed to rescue virulence of the Δ*rop18* knockout strain (Fig. 6C). These findings suggest that ROP5 and ROP18 play significant roles as type II virulence factors that resist host IRGs and parasite clearance, thereby promoting parasite survival and chronic infection. Surprisingly, ROP17 was associated with only a low-virulence phenotype, though this molecule was found to be as essential for chronic infection as ROP5 or ROP18. Moreover, parasites deficient in ROP35 or ROP38/29/19 were not virulence attenuated (Fig. 6E) yet were highly attenuated in cyst burdens (Fig. 1). Collectively, these findings suggest that additional mechanisms of host manipulation beyond direct interference with IRG functions at the PVM are associated with the successful development of chronic infection by type II strains of *T. gondii*.

## DISCUSSION

*Toxoplasma gondii* establishes a chronic infection of the host as an obligatory step for its biological transmission to new hosts (1). Herein, using the Δ*ku80* genetic background that enables efficient and reliable genetic dissection of parasite gene families (39, 40), we deleted 26 ROPK gene loci encoding 31 known ROPK proteins and show for the first time that many type II ROPK proteins influence the ability of *T. gondii* to establish chronic infection. Moreover, several ROPKs, including ROP5, ROP17, ROP18, ROP35, and ROP38/29/19 were found to provide crucial influences on functions that were necessary to establish chronic infection.

The roles of ROP5, ROP17, or ROP18 molecules in establishing chronic infection of the host have not been assessed previously. Type II strains carry a virulent *ROP18* gene allele and were previously proposed to behave in an avirulent fashion because type II strains also express avirulent *ROP5* gene alleles (16, 17, 26–28). We found that deletion of either type II ROP18 or ROP5 essentially abrogated chronic infection and markedly reduced virulence lethality by >100-fold. These results correlate with the significant defects found in the resistance of Δ*rop18* and Δ*rop5* PVs to IRG coating and killing, suggesting that ROP5 and ROP18 cooperate as type II virulence factors despite the absence of a highly virulent *ROP5* gene allele. Most likely, it is the absence of ROP5 gene alleles that limits type II virulence in comparison to the more highly virulent type I and South American strain types (15–17). This interpretation is consistent with previous evidence showing that coexpression of the virulent type I ROP5 gene locus in the type II background increased virulence by ~3 to 4 log units (28). Our study is the first to link the importance of type II ROP5 and ROP18 virulence functions to the development of cyst burdens and chronic infection.

Remarkably, nearly complete ablation of chronic infection in the Δ*rop5* and Δ*rop18* knockout strains was associated with only an ~10% reduction in parasite survival in IFN-γ-stimulated MEFs *in vitro*. Therefore, our results do not exclude the possibility that in addition to IRG resistance mechanisms, non-IRG functions of ROP5 and ROP18 complexes could also influence chronic infection. The N-terminal ROP18 RAH2 domain establishes PVM association, which is required for both IRG resistance and virulence but not for intrinsic ROP18 kinase activity (23, 24). The RAH2 domain also mediates ROP18 association with host ATF6β, and this interaction triggers the proteasome-dependent degradation of host ATF6β, and correspondingly, compromises adaptive immune responses mediated by CD8^+^ T cells (38). However, this specific ROP18-ATF6β interaction has only been previously demonstrated using recombinant ROP18 molecules that were not PVM associated (38). The multifunctional ROP18 molecule also associates with the p65 subunit of NF-κB to mediate its degradation (41). A second ROP18 ligand-binding pocket that influences virulence is also present in the ROP18 protein structure (42). Moreover, additional parasite proteins were recently identified in ROP5 and ROP18 complexes, suggesting there is unexpected biological complexity to their regulation and PVM-associated functions (22). Thus, future studies are necessary to decipher whether ROP5 and ROP18 IRG-independent mechanisms influence chronic infection.

Our findings establish that ROP17 is a key molecule that determines cyst development in type II strains. However, IRG resistance in the Δ*rop17* knockout strain was unaffected in IFN-γ-stimulated MEFs, and the Δ*rop17* strain exhibited only a low-virulence defect in comparison to deletion of ROP5 or ROP18. Previously, the deletion of ROP17 in the virulent type I RH strain was shown to reduce virulence, to increase Irgb6 coating of PVs, and to increase clearance of PVs in IFN-γ-stimulated macrophages (22). Together, these findings are consistent with previously reported evidence showing that the PVM-associated ROP17 kinase is polymorphic and under positive selective pressure in the virulent type I strain, which possesses 24-amino-acid differences compared with type II or type III ROP17 (22). Collectively, the data suggest that type II ROP17 is likely to play another role important for establishing chronic infection that is independent of the mild ROP17 virulence defect. The early egress phenotype of the Δ*rop17* knockout strain was not linked to an immediate defect in parasite invasion or replication, yet 13-day-old Δ*rop17* plaques were significantly reduced in their size. It is possible that ROP17 may function as a signaling molecule that influences the receptiveness of host cells to parasitism, the ability of the parasite to gain access to the central nervous system, or the development or maintenance of cyst burdens in the central nervous system. Recent elegant work based on rhoptry organelle secretion of *Cre* recombinase into host cells in contact with or invaded by *T. gondii* (43, 44) has demonstrated that ROP proteins are primarily injected into neurons within the brain (5). Another possibility is that the early egress phenotype of Δ*rop17* parasites may reduce the frequency of the overall success rate for tachyzoite differentiation to encysted bradyzoites *in vivo*. Alternatively, loss of ROP17 or other PVM-associated ROPK proteins may increase the susceptibility of the cyst wall to degradation by host chitinase, which is produced by alternatively activated macrophages in chronically infected mice (45). Future studies are necessary to more clearly identify the mechanisms that determine the key role of ROP17 in establishing chronic infection.

Severe reductions in cyst burdens were observed in the Δ*rop35* and Δ*rop38*/*29*/*19* knockout strains in the absence of any virulence defect *in vivo* or any defect in resistance to IRGs *in vitro*. These findings are intriguing in view of previous microarray evidence that defines ROP35 and ROP38 as the only *ROPK* genes that are significantly upregulated in less-virulent strain types (33). In particular, ROP38 transcripts are found to be more abundant in bradyzoite stages (increased >16-fold) compared to tachyzoite stages (33). Consequently, bradyzoite stage-specific expression of ROP38 may be important for the transition to or the maintenance of chronic stages *in vivo*. Because type II Δ*rop35* and Δ*rop39*/*29*/*19* knockout strains show severe impairments in cyst formation without changes in virulence, future studies may elucidate novel mechanisms that influence chronic infection.

While ROP38 expression is upregulated during bradyzoite stages, expression of ROP19 and ROP29, also present in the *ROP38*/*29*/*19* gene locus, is downregulated in the bradyzoite stage (33). Moreover, of 13 *ROPK* genes showing bradyzoite stage-specific transcriptional regulation *in vitro* (33), we found that deletion of 9 of these ROPKs (ROP4/7, ROP5, ROP17, ROP18, ROP19, ROP29, ROP38, ROP40, and ROP41) reduced cyst burdens, while deletion of ROP16 increased cyst burdens. Stage regulation of ROPK expression appears to be important for the development or maintenance of cyst burdens *in vivo*.

ROP35 is expressed in *Toxoplasma*, *Neospora*, *Eimeria*, and *Sarcocystis* and maps to a basal ROPK cluster designated ROPKL (34). ROP38-related genes are expanded in both *Toxoplasma* and cyst-forming *Neospora* coccidian parasites (33). *T. gondii* ROP38 strongly influences the expression of a large number of host genes involved in mitogen-activated protein kinase (MAPK) signaling cascades, apoptosis, and host cell proliferation (33). ROP16 is another ROPK that strongly influences the expression of a large number of host genes (8, 46, 47), and inhibits the responsiveness of its host cell to IFN-γ signaling (46). Knockdown of the host STAT1 transcriptional coactivator tailless (TLX) was recently shown to repress a subset of IFN-γ-activated genes in the brain, and mice lacking expression of TLX in the brain were found to be impaired in their ability to control chronic infection (48). These observations suggest that ROPK molecules, such as ROP38, ROP35, and ROP16, could influence chronic infection through regulation of host response genes. Our findings provide the first definitive evidence showing that the type II *T. gondii* rhoptry kinome provides anti-IRG virulence functions in acute infection as well as additional mechanisms of host manipulation necessary to establish chronic infection.

## MATERIALS AND METHODS

### Ethics statement.

All mouse work was conducted in accordance with the recommendations in the *Guide to the Care and Use of Laboratory Animals* (49) and Association for the Assessment and Accreditation of Laboratory Animal Care guidelines. The animal protocol was approved by the Dartmouth College Committee on the Use and Care of Animals (Animal Welfare Assurance A3259-01, protocol bzik.dj2). All efforts were made to minimize suffering and pain.

### Parasite culture.

Type II Prugniaud (Pru) background *Toxoplasma gondii* parasites were maintained by serial passage of tachyzoites in human foreskin fibroblast (HFF) monolayers cultured in Eagle’s modified essential medium (EMEM) containing 1% fetal bovine serum (FBS), 2 mM glutamine, 100 U/ml penicillin, and 100 µg/ml streptomycin as previously described (50, 51).

### Mice and generation of bone marrow-derived macrophages.

Female 7- to 9-week-old C57BL/6 mice were purchased from Jackson Laboratories (Bar Harbor, ME) and were maintained at the Center for Comparative Medicine and Research at the Geisel School of Medicine at Dartmouth. Bone marrow-derived macrophages were isolated from bone marrow from C57BL/6 mice by differentiation in Dulbecco’s modified essential medium (DMEM) supplemented with 10% FBS, 1× minimal essential medium nonessential amino acids, 1 mM sodium pyruvate (Life Technologies), antibiotics, and 30% L929 culture supernatant as previously described (52). Bone marrow-derived macrophages were harvested after 5 days of differentiation.

### Chronic infection.

Freshly lysed high-viability type II tachyzoites were obtained as previously described (11, 37). Tachyzoites were centrifuged at 900 × *g* for 7 min, washed, and counted in Dulbecco’s modified phosphate-buffered saline (DPBS). Mice were infected by intraperitoneal (i.p.) injection, and parasite viability was determined in a plaque assay at the time of mouse infection. To induce chronic infection, mice were infected i.p. with 2 × 10^2^ tachyzoites of the tested type II strain. Higher parasite doses (2 × 10^5^ or 2 × 10^6^) were used in certain experiments as indicated.

### Tissue cyst burden assay.

The brains from mice infected with type II strains were harvested at 3 weeks postinfection (11). The brains were homogenized in 2-ml volumes of sterile DPBS using a Dounce homogenizer. Cyst counts were performed on a minimum of 10% of each brain. Because Pru background cysts vary greatly in size and the brains of chronically infected mice contain many small, medium, and large cysts (11, 53), cysts were scored using dark-field microscopy with an inverted fluorescence phase-contrast microscope (Olympus CKX41) to reliably identify all cysts. The type II Δ*ku80* strain expresses green fluorescent protein (GFP) under control of the bradyzoite stage-specific *LDH2* promoter (11). GFP-positive (GFP^+^) cysts were scored at a total magnification of ×150 that provided the highest sensitivity for the detection of GFP^+^ bradyzoites within cysts and to then also verify the presence of a translucent thick cyst wall in bright-field microscopy (11, 37).

### Generation of *ROPK* knockout strains.

Deletion of ROP kinome gene loci was performed using the *KU80* knockout strain of the type II PruΔ*hxgprt* strain as previously described (11, 37). Briefly, gene locus knockout targeting plasmids were assembled in yeast shuttle vectors pRS416 or pRS426 using yeast recombinational cloning to fuse three distinct PCR products with 31- to 34-bp crossovers in order; a 5′ GOI (gene of interest) target gene flank, the *HXGPRT* selectable marker, and a 3′ GOI target flank (see Fig. S1A in the supplemental material) (36). Knockout plasmids were engineered to delete at least 200 nucleotides of the 5′ untranslated region (5′ UTR) and the complete coding region of the *ROPK* gene locus as defined in the ToxoDB.org database (54). All primers used to construct knockout targeting plasmids and nucleotide definition of *ROPK* gene loci deletions are listed in Table S2 in the supplemental material. After plasmid validation by DNA sequencing, targeting plasmids were linearized at restriction sites inserted at either the 5′ end of the 5′-targeting flank or at the 3′ end of the 3′-targeting flank. Linearized targeting plasmids were transfected by electroporation into tachyzoites of the PruΔ*ku80*Δ*hxgprt* strain and *ROPK* knockouts were selected in 50 µg/ml mycophenolic acid and 50 µg/ml xanthine, and parasites were cloned by limiting dilution 30 days after transfection. *ROPK* knockouts were validated by genotype analysis using PCR to measure the following (shown in Fig. S1A): (i) targeted deletion of the coding region of the targeted gene (DF and DR primers) in PCR 1, (ii) correct targeted 5′ integration (CXF and 5′DHFRCXR primers) in PCR 2, and (iii) correct targeted 3′ integration (3′DHFRCXF and CXR primers) in PCR 3 using knockout validation primers shown in Table S3.

### Complementation of ROPK knockouts.

Complementation plasmids were designed to complement *ROPK* knockout strains PruΔ*ku80*Δ*ropGOI* through chromosomal integration and expression of wild-type or mutant gene alleles at the uracil phosphoribosyltransferase (*UPRT*) chromosomal locus (TGME49_312480) as previously described (11) (see Fig. S1B in the supplemental material). Complementation plasmids were developed in the pRS416 or pRS426 yeast shuttle vector using yeast recombination to fuse, in order, a 5′ *UPRT* target flank, the complementing gene of interest with native 5′ UTR, and the 3′ *UPRT* target flank (Fig. S1B). Oligonucleotide DNA primers (Table S4) were used to generate the complementing genes, synthesized on one or two PCR products. ROP18 mutant gene alleles possessing a kinase-dead (KD) domain or deleted RAH2 domain [RAH2(ATF)] were engineered as previously described (19, 38). Following plasmid assembly by yeast recombinational cloning, targeting plasmids were validated by DNA sequencing. Prior to transfection, plasmids were linearized via the unique restriction site PmeI. The parasites were cultured for 2 days in normal infection media, and the cultures were then switched to selection medium containing 2 µM 5-fluorodeoxyuridine (FUDR) and cloned 30 days after transfection by limiting dilution. Targeting of complementing genes to the *UPRT* locus was validated by genotype analysis using PCR assays (strategy shown in Fig. S1B) to measure the following: (i) deletion of UPRT coding region in PCR 4, (ii) correct targeted 5′ integration in PCR 5, and (iii) correct targeted 3′ integration of the complementing transgene at the *UPRT* locus in PCR 6 using oligonucleotide DNA validation primers (Table S5).

### Characterization of *ROP5A* and *ROP5C* gene alleles.

Genomic DNA isolated from the Δ*ku80* parent strain was used as the template to PCR amplify the complete coding region of *ROP5A* and *ROP5C* gene alleles using primers ATGGCGACGAAGCTCGCTAGAC (forward) and GAGCCGTTTTCTCAAAGCGACTGAG (reverse). PCR products were randomly cloned into the pCR4 Topo vector (Invitrogen), and individual clones were sequenced. All sequences were managed with MacVector and aligned using ClustalW.

### Intracellular replication rate assay.

Parasite growth rate was determined using methods described previously employing a direct parasite per vacuole scoring approach (11). Briefly, triplicate monolayers of HFF cells were infected at a multiplicity of infection (MOI) of ~0.2. and parasites were allowed to invade cells for 1 h. Monolayers were then washed three times in DPBS to remove extracellular parasites. At 21 h and 45 h postinfection, tachyzoites per vacuole were scored in at least 50 randomly encountered vacuoles.

### Plaque assay.

Confluent monolayers of HFF cells were infected with 200 tachyzoites of a type II strain in infection medium, and cultures were left undisturbed for 13 days to allow localized development of PFU. The medium in the cultures was removed, and the cultures were fixed in 50% methanol and 10% acetic acid and stained with 0.25% Coomassie brilliant blue for 2 days. Cultures were rinsed in water, air dried, and photographed (55).

### Infection rate assay.

Parasite infection rate was determined in PFU assays. Briefly, multiple monolayers of HFF cells in 25-cm^2^ flasks were infected with 500 tachyzoites. At 30 and 60 min postinfection, the monolayers were washed three times in DPBS to remove extracellular parasites, and either infection medium was replaced, or parasites were not removed from continuously incubated control flask. The cultures were incubated continuously for 11 additional days to establish PFU. Percent infection rate was determined as the number of PFU present at each time divided by the number of PFU in the continuously incubated cultures.

### Early egress assay.

Tachyzoites in primary type II PruΔ*ku80* PVs egress at ~72 h postinfection (37). HFF cells were infected at an MOI of 0.01, and 1 h after infection, the monolayers were washed three times in DPBS to remove extracellular parasites, and infection medium was replaced. At least 500 infection sites were scored at 21, 45, 68, and 72 h postinfection to measure the percentage of intact primary PVs or secondary sites of infection sites (after parasite egress from the PV). The number of tachyzoites present in secondary infection sites was also measured at 45 h and 72 h postinfection by scoring at least 100 secondary sites of infection.

### PV killing assay.

C57BL/6 MEFs (ATCC) were seeded into 24-well trays and were cultured in DMEM in 15% FBS. MEF monolayers were incubated without IFN-γ, or MEF monolayers were stimulated with 200 U/ml IFN-γ (Peprotech) 24 h prior to parasite infection to activate host immunity-related GTPases and cell autonomous killing mechanisms. Triplicate wells of MEFs were infected with 200 or 1,000 tachyzoites, and plaques were allowed to develop for 5 to 7 days. The total number of PFU per well was counted microscopically, and the percentage of PFU survival was calculated as the number of PFU in IFN-γ-stimulated MEFs divided by the number of PFU scored in nonstimulated MEFs (no IFN-γ treatment).

### IRG coating assay.

Bone marrow-derived macrophages were harvested, seeded on circular cover glasses (Electron Microscopy Sciences), and incubated overnight, and then macrophages were primed with 100 U/ml IFN-γ and 10 U/ml tumor necrosis factor alpha (TNF-α) (Peprotech) for 6 h. The macrophages on the coverslips were infected with parasites at an MOI of 4 for 45 min, the coverslips were washed in DPBS, and infected cells were fixed with 4% paraformaldehyde (Electron Microscopy Sciences), permeabilized with 0.1% saponin (Sigma), and blocked in 10% FBS. For visualization, cultures were incubated with mouse anti-GRA5 (1:2,000) (clone TG 17.113; Biotem) and rabbit anti-Irgb6 (1:1,000) (56), then washed and incubated with secondary antibodies anti-mouse antibodies conjugated to Alexa Fluor 568 and anti-rabbit antibodies conjugated to Alexa Fluor 488 (Invitrogen). Coverslips were mounted in ProLong gold with DAPI (4′,6′-diamidino-2-phenylindole) (Invitrogen) and imaged at 63× with a Nikon A1R SI confocal microscope (Nikon, Inc.). All images were processed with FIJI (57). A minimum of 500 PVs was scored for each strain for quantification of Irgb6 PV coating.

### Immunofluorescence assay.

HFF cells were cultured on circular microcover glasses and infected with parasites for ~16 to 30 h. To visualize ROP5, the cultures were fixed in 4% paraformaldehyde and permeabilized with 0.01% Triton X-100. To visualize ROP17 and ROP18, the cultures were fixed with Histochoice (Amresco) and permeabilized in 0.1% saponin (Sigma) for 10 min. All samples were blocked with 10% FBS and incubated with primary antibodies for 1 h at room temperature (RT). The primary antibodies were rabbit anti-ROP5 (MO556; 1:3,000 dilution) (26), rabbit anti-ROP18 (ROP18-His WA525; 1:500 dilution; Sibley Laboratory), and anti-HA to visualize ROP17^FLHA^ (rabbit monoclonal anti-HA tag antibodies; 1:500 dilution; Cell Signaling). Preparations were washed three times with PBS and incubated 1 h at RT with a 1:1,000 dilution of secondary goat anti-rabbit IgG antibodies conjugated to Alexa Fluor 488. Samples were mounted in SlowFade gold with DAPI (Life Technologies).

### *In vitro* cyst differentiation assay.

Tachyzoites were differentiated *in vitro* into bradyzoites within cysts essentially as previously and elegantly described by Tobin and colleagues (58). Differentiation media contained Roswell Park Memorial Institute medium (RPMI) without bicarbonate supplemented with 2.05 mM l-glutamine (Hyclone), 20 mM HEPES-free acid (IBI Scientific), 1% XL-glutamine (a long-lasting stable form of glutamine; VWR), 1% FBS, and 1% penicillin-streptomycin. The pH of differentiation medium was adjusted to 8.1 with sodium hydroxide and filter sterilized. HFF cells were cultured on circular microcover glass, and confluent monolayers were infected with type II parasites at an MOI of ~0.5. Three hours after infection, the infected cells were washed once in DPBS supplemented with Ca^2+^ and Mg^2+^, and cultures were incubated in differentiation media for 3 days at 37°C in ambient air. Infected cells were fixed in 4% paraformaldehyde, and the excess was quenched with 0.1 M glycine. All samples were permeabilized and blocked in 3% FBS plus 0.2% Triton X-100 for 30 min at room temperature, and then they were incubated with a 1:250 dilution of rhodamine-labeled *Dolichos biflorus* agglutinin (Vector Laboratories) for 1 h at RT. The preparations were washed three times with DPBS, mounted in SlowFade gold antifade with DAPI (Life Technologies), and imaged by confocal microscopy.

## SUPPLEMENTAL MATERIAL

Figure S1  Knockout and complementation strategy. (A) Knockout strategy to insert *HXGPRT* at deleted gene loci. The design of genotype validation PCR is shown. MPA, mycophenolic acid; X, xanthine. B) Complementation strategy at the UPRT locus. The design of genotype validation PCR is shown. FUDR, 5-fluorodeoxyuridine. Download Figure S1, TIF file, 0.3 MB

Figure S2  Complementation of Δ*rop5*, Δ*rop17*, and Δ*rop18*. Wild-type or mutant gene alleles or ROP5, ROP17, or ROP18, as designated, were evaluated for expression and rhoptry localization of protein products. Nuclei were stained with DAPI, and PVs were identified using DIC microscopy. Download Figure S2, TIF file, 1.5 MB

Figure S3  Amino acid sequence of type II *ROP5* alleles. Type II *ROP5* gene alleles were characterized by gene sequencing and compared with the corresponding *ROP5* gene alleles of strain ME49. *ROP5A* allele-specific amino acid changes are shown in green, and *ROP5C* allele-specific amino acid changes are shown in red. Download Figure S3, TIF file, 2.2 MB

Figure S4  Intracellular replication rates of *ROPK* knockouts that influence chronic infection. (A) Replication rate, measured as the average number of tachyzoites per vacuole 45 h postinfection, was determined for *ROPK* knockout strains that exhibited reductions or increases in cyst burdens. (B) The percentage of parasite vacuoles containing various number of tachyzoites was measured 45 h postinfection for the *Δrop17* and *Δrop17*::*ROP17^FLHA^* knockout strains and the parental *Δku80* strain. (C) The number of tachyzoites present in secondary sites of infection was determined at 45 h and 72 h postinfection for the *Δrop17* and *Δrop17*::*ROP17^FLHA^* strains and the parental *Δku80* strain. Download Figure S4, TIF file, 0.4 MB

Table S1  Genotypes of strains developed or used in this study. Table S1, DOC file, 0.1 MB

Table S2  Primers used to construct *ROPK* knockouts. The sequences of oligonucleotide forward primers (FP) and reverse primers (RP) used for construction of deletion-targeting plasmids pROP2/8P (Δ*rop2*/*8*), pROP5P (Δ*rop5*), pROP11P (Δ*rop11*), pROP16P (Δ*rop16*), pROP17P (Δ*rop17*), pROP18P (Δ*rop18*), pROP20P (Δ*rop20*), pROP21P (Δ*rop21*), pROP22P (Δ*rop22*), pROP23P (Δ*rop23*), pROP24P (Δ*rop24*), pROP25P (Δ*rop25*), pROP26P (Δ*rop26*), pROP27P (Δ*rop27*), pROP28P (Δ*rop28*), pROP30P (Δ*rop30*), pROP31P (Δ*rop31*), pROP32P (Δ*rop32*), pROP35P (Δ*rop35*), pROP36P (Δ*rop36*), pROP37P (Δ*rop37*), and pROP38P (Δ*rop38*/*29*/*19*), pROP39P (Δ*rop39*), pROP40P (Δ*rop40*), pROP41P (Δ*rop41*), pROP42,43,44P (Δ*rop42*/*43*/*44*), and pROP45P (Δ*rop45*) are shown. The corresponding *T*. *gondii* ME49 (TgME49) gene locus, chromosome, and nucleotides deleted for each knockout were determined from data in toxodb.org. Table S2, DOCX file, 0.1 MB

Table S3  Primers used to validate *ROPK* knockouts. The sequences of oligonucleotide primers used for validation of Δ*rop2*/*8*, Δ*rop5*, Δ*rop11*, Δ*rop16*, Δ*rop17*, Δ*rop18*, Δ*rop20*, Δ*rop21*, Δ*rop22*, Δ*rop23*, Δ*rop24*, Δ*rop25*, Δ*rop26*, Δ*rop27*, Δ*rop28*, Δ*rop30*, Δ*rop31*, Δ*rop32*, Δ*rop35*, Δ*rop36*, Δ*rop37*, Δ*rop38*/*29*/*19*, Δ*rop39*, Δ*rop40*, Δ*rop41*, Δ*rop42*/*43*/*44*, and Δ*rop45* knockouts are shown. The primers were designed from TgME49 data in toxodb.org. Table S3, DOCX file, 0.03 MB

Table S4  Primers used to develop targeting plasmids for complementation. The sequences of oligonucleotide forward primers (FP) and reverse primers (RP) used for construction of Pru complementation vectors pURO5A (::*ROP5A*), pURO5C (::*ROP5C*), pURO17^FLHA^ (::*ROP17^FLHA^*), pURO18 (::*ROP18*), pURO18^KD^ (::*ROP18^KD^*), and pURO18^*RAH2*(*ATF*)^ [::*ROP18*^RAH2(*ATF*)^] are shown. The corresponding TgME49 gene locus, chromosome, and nucleotides deleted for each knockout were determined from data in toxodb.org. Table S4, DOCX file, 0.04 MB

Table S5  Primers used to validate complementation of *ROPK* knockouts. The sequences of oligonucleotide primers used to validate integration of complementation vectors pURO5A (::*ROP5A*), pURO5C (::*ROP5C*), pURO17^FLHA^ (::*ROP17^FLHA^*), pURO18 (::*ROP18*), pURO18^KD^ (::*ROP18^KD^*), and pURO18^*RAH2*(*ATF*)^ [::*ROP18*^RAH2(*ATF*)^] are shown. Primers were designed from TgME49 data in toxodb.org. Table S5, DOCX file, 0.02 MB
