# Supplementary figures and images for: The Toxoplasma gondii Rhoptry Kinome Is Essential for Chronic Infection
Source: mBio. 2016 May 10;7(3):e00193-16. doi: 10.1128/mBio.00193-16 (PMC4959664; doi:10.1128/mBio.00193-16)

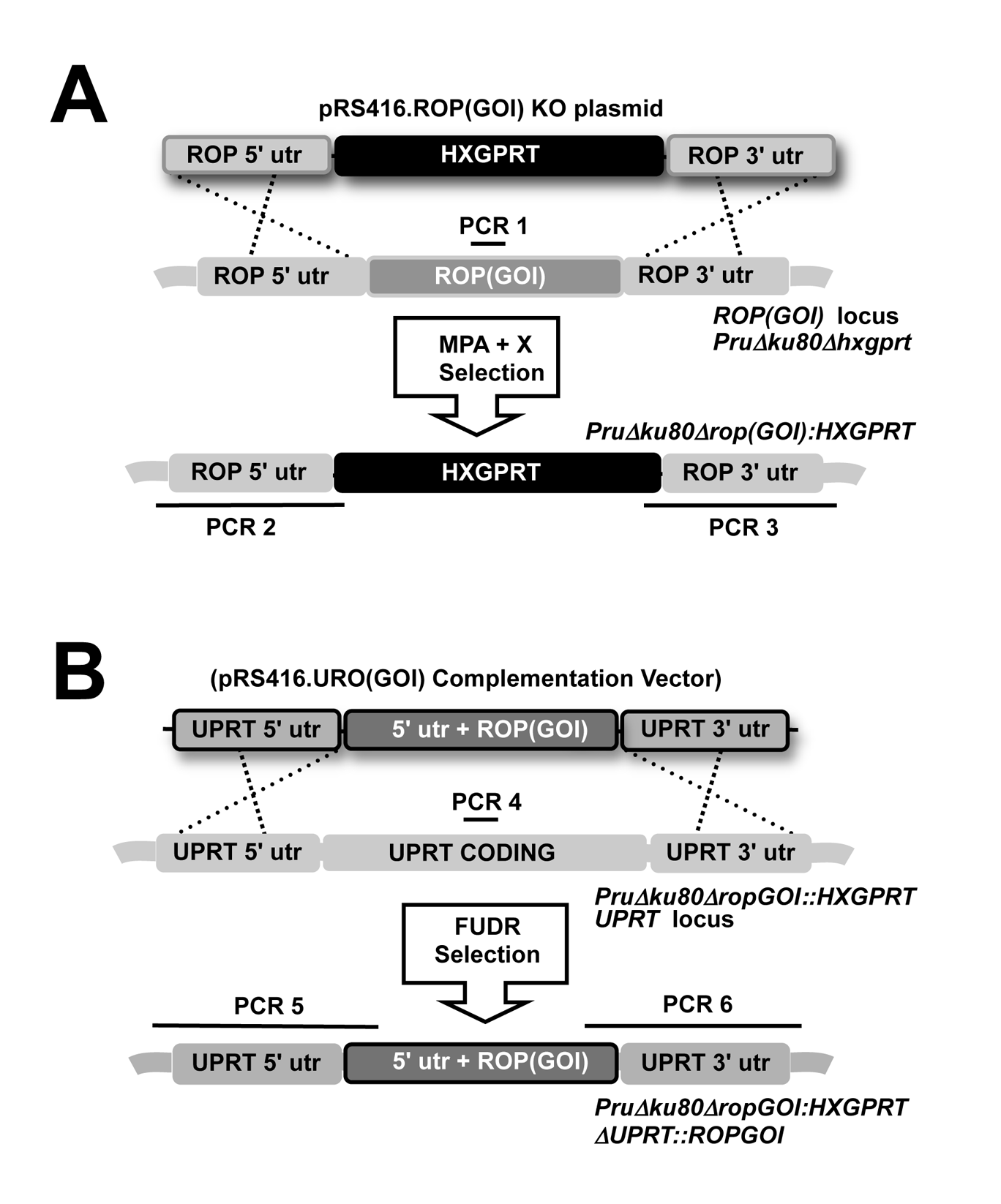

Supplement: Figure S1 — Knockout and complementation strategy. (A) Knockout strategy to insert HXGPRT at deleted gene loci. The design of genotype validation PCR is shown. MPA, mycophenolic acid; X, xanthine. B) Complementation strategy at the UPRT locus. The design of genotype validation PCR is shown. FUDR, 5-fluorodeoxyuridine. Download [file mbo002162811sf1.tif]

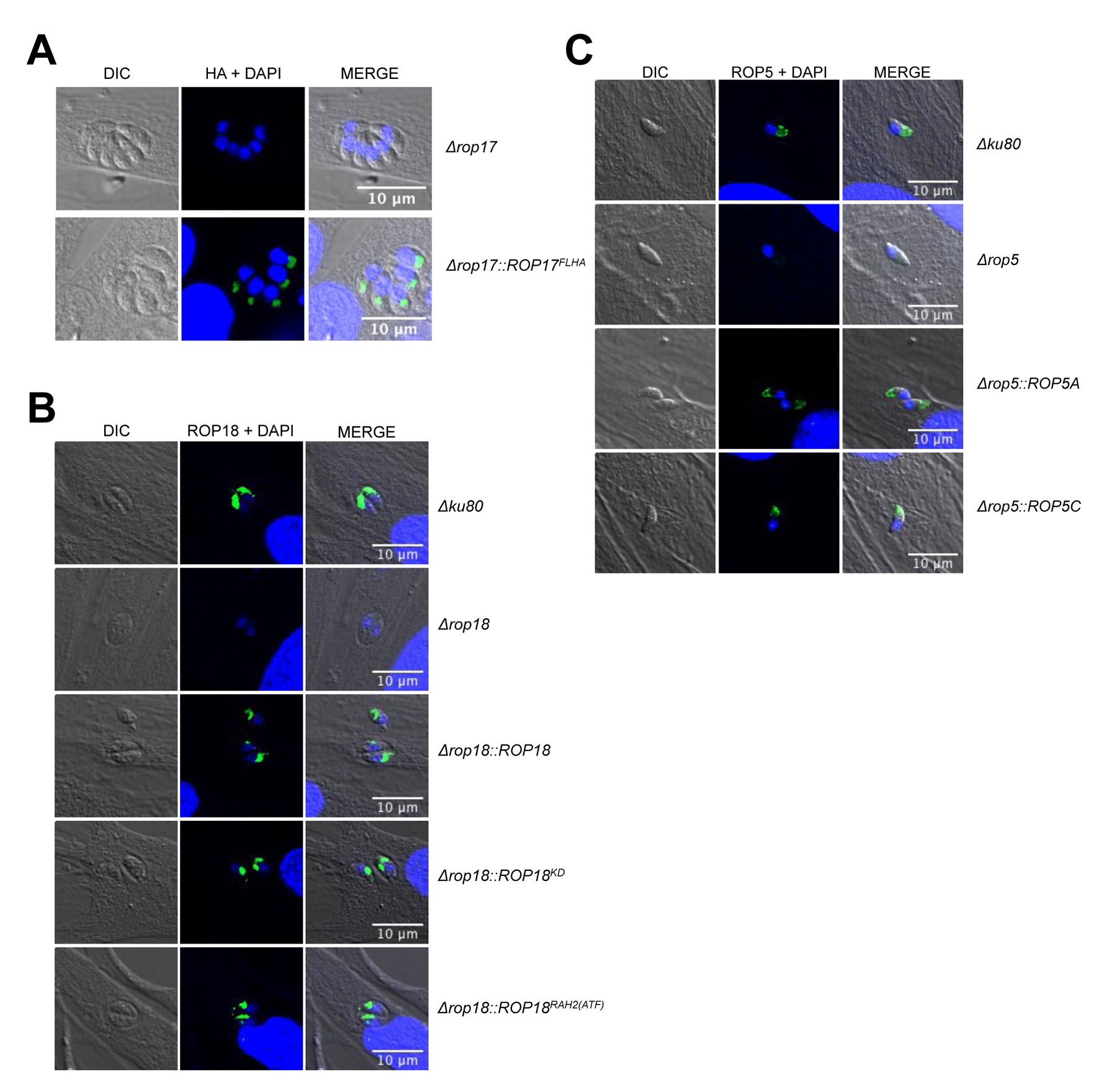

Supplement: Figure S2 — Complementation of Δrop5, Δrop17, and Δrop18. Wild-type or mutant gene alleles or ROP5, ROP17, or ROP18, as designated, were evaluated for expression and rhoptry localization of protein products. Nuclei were stained with DAPI, and PVs were identified using DIC microscopy. Download [file mbo002162811sf2.tif]

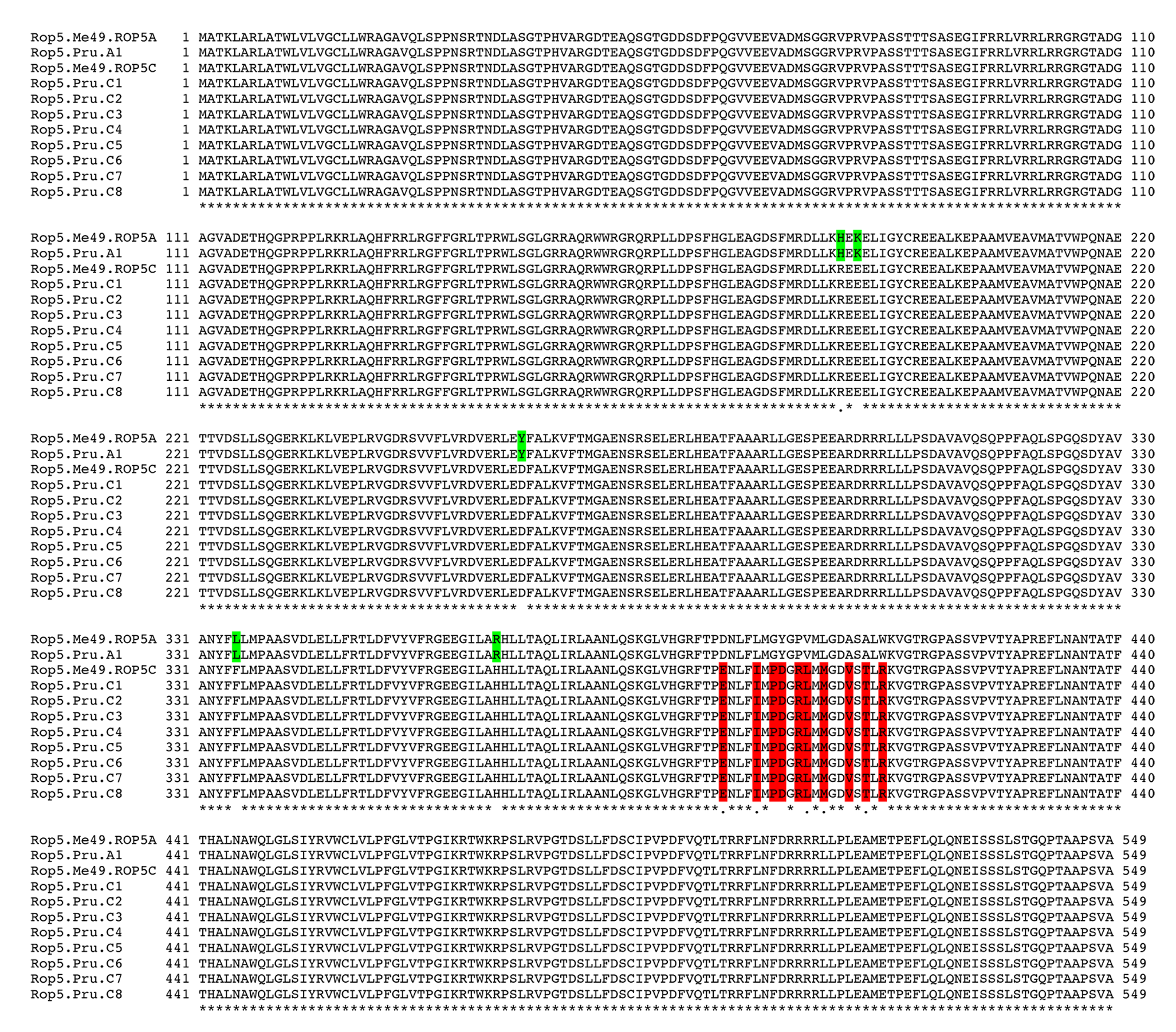

Supplement: Figure S3 — Amino acid sequence of type II ROP5 alleles. Type II ROP5 gene alleles were characterized by gene sequencing and compared with the corresponding ROP5 gene alleles of strain ME49. ROP5A allele-specific amino acid changes are shown in green, and ROP5C allele-specific amino acid changes are shown in red. Download [file mbo002162811sf3.tif]

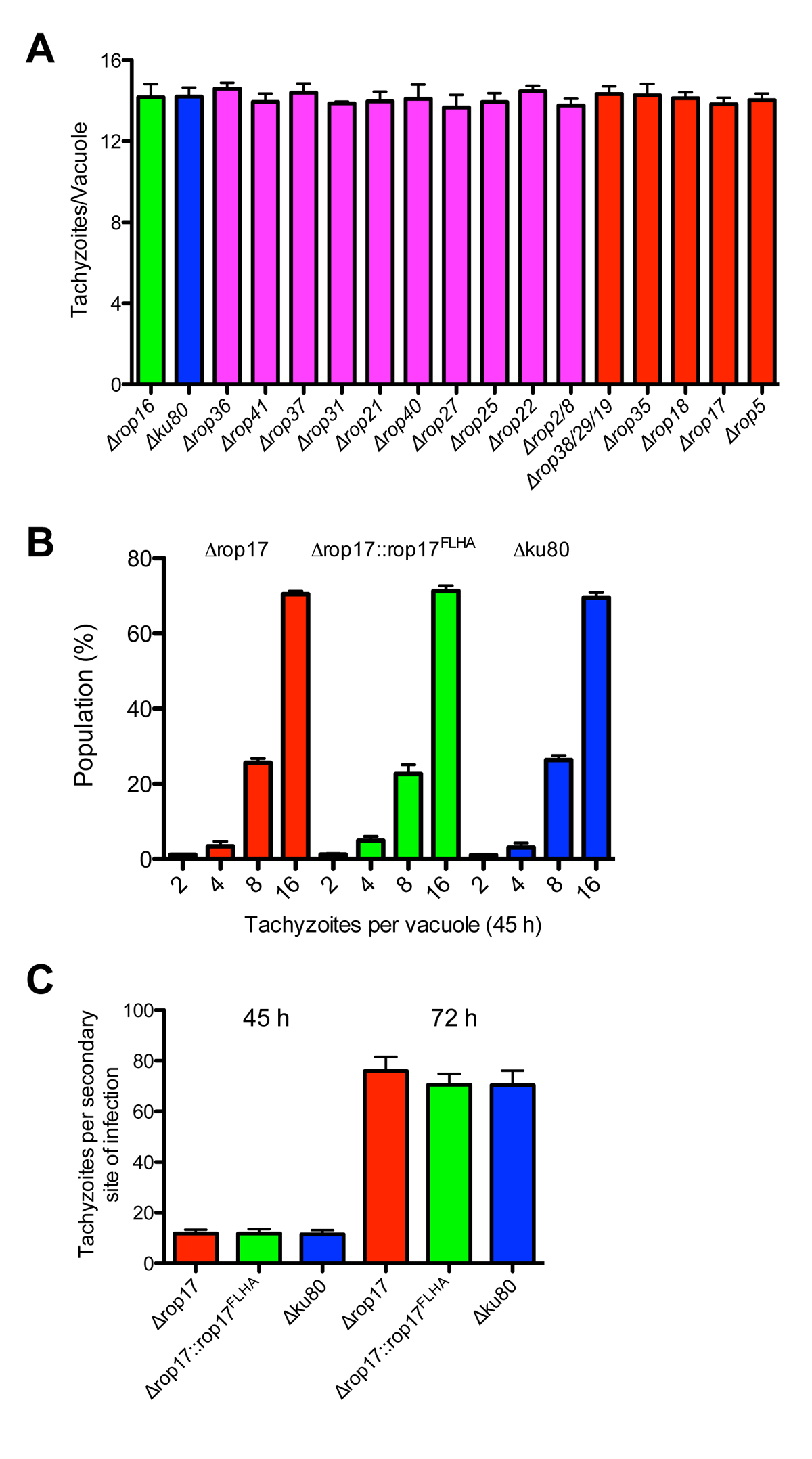

Supplement: Figure S4 — Intracellular replication rates of ROPK knockouts that influence chronic infection. (A) Replication rate, measured as the average number of tachyzoites per vacuole 45 h postinfection, was determined for ROPK knockout strains that exhibited reductions or increases in cyst burdens. (B) The percentage of parasite vacuoles containing various number of tachyzoites was measured 45 h postinfection for the Δrop17 and Δrop17::ROP17FLHA knockout strains and the parental Δku80 strain. (C) The number of tachyzoites present in secondary sites of infection was determined at 45 h and 72 h postinfection for the Δrop17 and Δrop17::ROP17FLHA strains and the parental Δku80 strain. Download [file mbo002162811sf4.tif]
