# Supplementary material for: The Toxoplasma gondii Rhoptry Kinome Is Essential for Chronic Infection
Source: mBio. 2016 May 10;7(3):e00193-16. doi: 10.1128/mBio.00193-16 (PMC4959664; doi:10.1128/mBio.00193-16)
Supplement: Table S1 — Genotypes of strains developed or used in this study. [file mbo002162811st1.doc]

**Table S1. Genotypes of strains used or developed in this study.**

| **Strain designation** | **ToxoDB (corresponding TGME49 locus)** | **Strain genotype** | **Source** |
| --- | --- | --- | --- |
| Δ*ku80* |  | PruΔ*ku80*Δ*hxgprt* | (1) |
| Δ*rop2/8* | TGME49_215750, 215775 | PruΔ*ku80*Δ*hxgprt*Δ*rop2A/2B/8HXGPRT* | (This paper) |
| Δ*rop4/7* | TGME49_295125, 295110, 295105 | PruΔ*ku80*Δ*hxgprt*Δ*rop4/7A/7B/7CHXGPRT* | (1) |
| Δ*rop5* | TGME49_308075, 308090, to 308096 | PruΔ*ku80*Δ*hxgprt*Δ*rop5HXGPRT* | (This paper) |
| Δ*rop5::ROP5A* | TGME49_308096 | PruΔ*ku80*Δ*hxgprt*Δ*rop5HXGPRT*Δ*uprt::ROP5A* | (This paper) |
| Δ*rop5ROP5C* | TGME49_308090 | PruΔ*ku80*Δ*hxgprt*Δ*rop5HXGPRT*Δ*uprt::ROP5C* | (This paper) |
| Δ*rop11* | TGME49_227810 | PruΔ*ku80*Δ*hxgprt*Δ*rop11HXGPRT* | (This paper) |
| Δ*rop16* | TGME49_262730 | PruΔ*ku80*Δ*hxgprt*Δ*rop16HXGPRT* | (This paper) |
| Δ*rop17* | TGME49_258580 | PruΔ*ku80*Δ*hxgprt*Δ*rop17HXGPRT* | (This paper) |
| Δ*rop17::ROP17^FLHA^* | TGME49_258580 | PruΔ*ku80*Δ*hxgprt*Δ*rop17HXGPRT*Δ*uprt::ROP17^FLHA^* | (This paper) |
| Δ*rop18* | TGME49_205250 | PruΔ*ku80*Δ*hxgprt*Δ*rop18HXGPRT* | (This paper) |
| Δ*rop18ROP18* | TGME49_205250 | PruΔ*ku80*Δ*hxgprt*Δ*rop18::HXGPRT*Δ*uprt::ROP18* | (This paper) |
| Δ*rop18ROP18ATF* | TGME49_205250 | PruΔ*ku80*Δ*hxgprt*Δ*rop18*::*HXGPRT*Δ*uprt::ROP18ATF* | (This paper) |
| Δ*rop18ROP18* | TGME49_205250 | PruΔ*ku80*Δ*hxgprt*Δ*rop18*::*HXGPRT*Δ*uprt::ROP18* | (This paper) |
| Δ*rop20* | TGME49_258230 | PruΔ*ku80*Δ*hxgprt*Δ*rop20HXGPRT* | (This paper) |
| Δ*rop21* | TGME49_263220 | PruΔ*ku80*Δ*hxgprt*Δ*rop21HXGPRT* | (This paper) |
| Δ*rop22* | TGME49_207700 | PruΔ*ku80*Δ*hxgprt*Δ*rop22HXGPRT* | (This paper) |
| Δ*rop23* | TGME49_239600 | PruΔ*ku80*Δ*hxgprt*Δ*rop23HXGPRT* | (This paper) |
| Δ*rop24* | TGME49_252360 | PruΔ*ku80*Δ*hxgprt*Δ*rop24HXGPRT* | (This paper) |
| Δ*rop25* | TGME49_202780 | PruΔ*ku80*Δ*hxgprt*Δ*rop25HXGPRT* | (This paper) |
| Δ*rop26* | TGME49_211260 | PruΔ*ku80*Δ*hxgprt*Δ*rop26HXGPRT* | (This paper) |
| Δ*rop27* | TGME49_313330 | PruΔ*ku80*Δ*hxgprt*Δ*rop27HXGPRT* | (This paper) |
| Δ*rop28* | TGME49_258370 | PruΔ*ku80*Δ*hxgprt*Δ*rop28HXGPRT* | (This paper) |
| Δ*rop31* | TGME49_258800 | PruΔ*ku80*Δ*hxgprt*Δ*rop31HXGPRT* | (This paper) |
| Δ*rop32* | TGME49_270920 | PruΔ*ku80*Δ*hxgprt*Δ*rop32HXGPRT* | (This paper) |
| Δ*rop35* | TGME49_304740 | PruΔ*ku80*Δ*hxgprt*Δ*rop35HXGPRT* | (This paper) |
| Δ*rop36* | TGME49_207610 | PruΔ*ku80*Δ*hxgprt*Δ*rop36::* | (This paper) |
| Δ*rop37* | TGME49_294560 | PruΔ*ku80*Δ*hxgprt*Δ*rop37::* | (This paper) |
| Δ*rop38/29/19* | TGME49_242100, 242110, 242230, 24240, 242250 | PruΔ*ku80*Δ*hxgprt*Δ*rop38*/*29*/*19A*/*19BHXGPRT* | (This paper) |
| Δ*rop39* | TGME49_262050 | PruΔ*ku80*Δ*hxgprt*Δ*rop39HXGPRT* | (This paper) |
| Δ*rop40* | TGME49_291960 | PruΔ*ku80*Δ*hxgprt*Δ*rop40HXGPRT* | (This paper) |
| Δ*rop41* | TGME49_266100 | PruΔ*ku80*Δ*hxgprt*Δ*rop41::HXGPRT* | (This paper) |
| Δ*rop42*/*43*/*44* | TGME49_209985, 210095, 210100, 321710, 321700 | PruΔ*ku80*Δ*hxgprt*Δ*rop42*/*43*/*44HXGPRT* | (This paper) |
| Δ*rop45* | TGME49_281675 | PruΔ*ku80*Δ*hxgprt*Δ*rop45::HXGPRT* | (This paper) |

**SUPPLEMENTAL REFERENCES**

1. **Fox BA, Falla A, Rommereim LM, Tomita T, Gigley JP, Mercier C, Cesbron-Delauw MF, Weiss LM, Bzik DJ.** 2011. Type II Toxoplasma gondii KU80 knockout strains enable functional analysis of genes required for cyst development and latent infection. Eukaryot Cell **10:**1193-1206.
