# Supplementary material for: The Toxoplasma gondii Rhoptry Kinome Is Essential for Chronic Infection
Source: mBio. 2016 May 10;7(3):e00193-16. doi: 10.1128/mBio.00193-16 (PMC4959664; doi:10.1128/mBio.00193-16)
Supplement: Table S2 — Primers used to construct ROPK knockouts. The sequences of oligonucleotide forward primers (FP) and reverse primers (RP) used for construction of deletion-targeting plasmids pROP2/8P (Δrop2/8), pROP5P (Δrop5), pROP11P (Δrop11), pROP16P (Δrop16), pROP17P (Δrop17), pROP18P (Δrop18), pROP20P (Δrop20), pROP21P (Δrop21), pROP22P (Δrop22), pROP23P (Δrop23), pROP24P (Δrop24), pROP25P (Δrop25), pROP26P (Δrop26), pROP27P (Δrop27), pROP28P (Δrop28), pROP30P (Δrop30), pROP31P (Δrop31), pROP32P (Δrop32), pROP35P (Δrop35), pROP36P (Δrop36), pROP37P (Δrop37), and pROP38P (Δrop38/29/19), pROP39P (Δrop39), pROP40P (Δrop40), pROP41P (Δrop41), pROP42,43,44P (Δrop42/43/44), and pROP45P (Δrop45) are shown. The corresponding T. gondii ME49 (TgME49) gene locus, chromosome, and nucleotides deleted for each knockout were determined from data in toxodb.org. [file mbo002162811st2.docx]

**Table S2. Primers used to construct *ROPK* knockouts.**

**Primer Primer Sequence Primer Use KO Construct Corresponding TGME49 locus**

**PMiniHXF** GATAAGCTTGATCAGCACGAAACCTTG HXGPRT cassette FP HXGPRT mini cassette

**PMiniHXR** CCGCTCTAGAACTAGTGGATCCC HXGPRT cassette RP

**ROP2/8F1** *TTGGGTAACGCCAGGGTTTTCCCAGTCACGACG*GTTTAAAC**CGTCTGCCTGAGCAGTTCTGTC** Pru ROP2/8 KO 5’ target FP pRS416.ROP2/8P TGME49_215775 chrX 7,151,817 to 7,161,568 (+)

**ROP2/8R1** *GCGGGTTTGAATGCAAGGTTTCGTGCTGATCAA*ACTAGT**GTGTGCAGAGTCGGACTAACGC** Pru ROP2/8 KO 5’ target RP

**ROP2/8F2** *TTCTGGCAGGCTACAGTGACACCGCGGTGGAGG*ACTAGT**CAGGAGTTATGAGTGCGCCTCAAG** Pru ROP2/8 KO 3’ target FP

**ROP2/8R2** *GTGAGCGGATAACAATTTCACACAGGAAACAGC*GCGGCCGC**CAAGCCAGTAGTGGATGTCGCAC** Pru ROP2/8 KO 3’ target RP

**ROP5F1** *TTGGGTAACGCCAGGGTTTTCCCAGTCACGACG*GTTTAAAC**GAGAAGATACTGATGTGCTGCACACG** Pru ROP5 KO 5’ target FP pRS426.ROP5P TGME49_308090 chrXII 564,553 to 577,981 (-)

**ROP5R1** *GCGGGTTTGAATGCAAGGTTTCGTGCTGATCAA*ACTAGT**GGCATTGCAGAATCTATGCAGCCAG** Pru ROP5 KO 5’ target RP

**ROP5F2** *TTCTGGCAGGCTACAGTGACACCGCGGTGGAGG*ACTAGT**TCCACTCACTGGTGTAGTCGATGC** Pru ROP5 KO 3’ target FP

**ROP5R2** *GTGAGCGGATAACAATTTCACACAGGAAACAGC*GCGGCCGC**GTGTAGCGTGCCACACTTCGC** Pru ROP5 KO 3’ target RP

**ROP11F1** *TTGGGTAACGCCAGGGTTTTCCCAGTCACGACG*GTTTAAAC**GATGCCAAGGTGAACACGCGC** Pru ROP11 KO 5’ target FP pRS416.ROP11P TGME49_227810 chrX 805,543 to 807,927 (+)

**ROP11R1** *GCGGGTTTGAATGCAAGGTTTCGTGCTGATCAA*ACTAGT**CGCTTGAGATCTGCGGAACGC** Pru ROP11 KO 5’ target RP

**ROP11PF2** *TTCTGGCAGGCTACAGTGACACCGCGGTGGAGG*ACTAGT**TGACGCAGTCACAGCTACCGAC** Pru ROP11 KO 3’ target FP

**ROP11R2** *GTGAGCGGATAACAATTTCACACAGGAAACAGC*GCGGCCGC**TGTCCGATGGCGGAATCGTACG** Pru ROP11 KO 3’ target RP

**ROP16F1** *TTGGGTAACGCCAGGGTTTTCCCAGTCACGACG*GTTTAAAC**GGCGTTCTGTGTTAGCTGCCAG** Pru ROP16 KO 5’ target FP pRS416.ROP16P TGME49_262730 chrVIIb 1,054,385 to 1,056,583 (-)

**ROP16R1** *GCGGGTTTGAATGCAAGGTTTCGTGCTGATCAA*ACTAGT**CATAGGCACTACCAGTGGTGCATTG** Pru ROP16 KO 5’ target RP

**ROP16F2** *TTCTGGCAGGCTACAGTGACACCGCGGTGGAGG*ACTAGT**CGAATCTGATCCAGCAGTGATGGG** Pru ROP16 KO 3’ target FP

**ROP16R2** *GTGAGCGGATAACAATTTCACACAGGAAACAGC*GCGGCCGC**CCCATGTCTCTTAAGGTGTGCGTC** Pru ROP16 KO 3’ target RP

**ROP17F1** *TTGGGTAACGCCAGGGTTTTCCCAGTCACGACG*GTTTAAAC**GCCGGACTGTAACCCGAAGC** Pru ROP17 KO 5’ target FP pRS416.ROP17P TGME49_258580 chrVIIb 3,321,656 to 3,324,424 (-)

**ROP17R1** *GCGGGTTTGAATGCAAGGTTTCGTGCTGATCAA*TCTAGA**AGACTGGTGCAGCTGGCCTG** Pru ROP17 KO 5’ target RP

**ROP17F2** *TTCTGGCAGGCTACAGTGACACCGCGGTGGAGG*TCTAGA**GACTACGCCACATGTACCACTCG** Pru ROP17 KO 3’ target FP

**ROP17R2** *GTGAGCGGATAACAATTTCACACAGGAAACAGC*GCGGCCGC**AGGCGACTCCGTCAGTCTTCC** Pru ROP17 KO 3’ target RP

**ROP18F1** TTGGGTAACGCCAGGGTTTTCCCAGTCACGACGGTTTAAAC**GGAACTCTGATTGAACCTGCGTG** Pru ROP18 KO 5’ target FP pRS416.ROP18P TGME49_205250 chrVIIa 1,514,558 to 1,516,300 (-)

**ROP18R1** GCGGGTTTGAATGCAAGGTTTCGTGCTGATCAATCTAGA**AGAGGTGCATAGCGTGGCTAGC** Pru ROP18 KO 5’ target RP

**ROP18F2**  TTCTGGCAGGCTACAGTGACACCGCGGTGGAGGTCTAGA**GATGCTCCCGCTACAAGCCTTG** Pru ROP18 KO 3’ target FP

**ROP18PR2** GTGAGCGGATAACAATTTCACACAGGAAACAGCGCGGCCGC**TGCAGAGTCGATACGAACAACATGG** Pru ROP18 KO 3’ target RP

**ROP20F1** *TTGGGTAACGCCAGGGTTTTCCCAGTCACGACG*GTTTAAAC**CCGTTGGCTCAAATACCTCGAGC** Pru ROP20 KO 5’ target FP pRS416.ROP20P TGME49_258230 chrVIIb 3,491,730 to 3,493,717 (-)

**ROP20R1** *GCGGGTTTGAATGCAAGGTTTCGTGCTGATCAA*ACTAGT**GGGCAAGCCAAGCAACTCAGG** Pru ROP20 KO 5’ target RP

**ROP20F2** *TTCTGGCAGGCTACAGTGACACCGCGGTGGAGG*ACTAGT**CGCTGCAAGGCCTTGGATCTG** Pru ROP20 KO 3’ target FP

**ROP20R2** *GTGAGCGGATAACAATTTCACACAGGAAACAGC*GCGGCCGC**AGGCGCAGAGTAGTGGATAAGCC** Pru ROP20 KO 3’ target RP

**ROP21F1** *TTGGGTAACGCCAGGGTTTTCCCAGTCACGACG*GTTTAAAC**GTCACACCAAGCACTGGGATGC** Pru ROP21KO 5’ target FP pRS416.ROP21P TGME49_ 263220 chrVIIb 699,468 to 704,968 (+)

**ROP21R1** *GCGGGTTTGAATGCAAGGTTTCGTGCTGATCAA*TCTAGA**CAACACTGCGGCGACTTACGG** Pru ROP21KO 5’ target RP

**ROP21F2** *TTCTGGCAGGCTACAGTGACACCGCGGTGGAGG*TCTAGA**GCAAGCAGGCAGCGACATCG** Pru ROP21KO 3’ target FP

**ROP21R2** *GTGAGCGGATAACAATTTCACACAGGAAACAGC*GCGGCCGC**TCCCAAACGTGTTCCAATAGTCCG** Pru ROP21KO 3’ target RP

**ROP22F1** *TTGGGTAACGCCAGGGTTTTCCCAGTCACGACG*GTTTAAAC**ACCTGTCGGGAGGAAGATGTGC** Pru ROP22 KO 5’ target FP pRS416.ROP22P TGME49_207700 chrIb 218,687 to 220,545 (-)

**ROP22R1** *GCGGGTTTGAATGCAAGGTTTCGTGCTGATCAA*ACTAGT**CTGATAGGTATTGCAAGGCGCGC** Pru ROP22 KO 5’ target RP

**ROP22F2** *TTCTGGCAGGCTACAGTGACACCGCGGTGGAGG*ACTAGT**CCTGCGAACCGGATCACTTCG** Pru ROP22 KO 3’ target FP

**ROP22R2** *GTGAGCGGATAACAATTTCACACAGGAAACAGC*GCGGCCGC**CTGTGGTGGGCTTGTCACTTCC** Pru ROP22KO 3’ target RP

**ROP23F1** *TTGGGTAACGCCAGGGTTTTCCCAGTCACGACG*GTTTAAAC**GAGAAAACATAGTGGCGCCGGC** Pru ROP23 KO 5’ target FP pRS416.ROP23P TGME49_239600 chrVI 793,940 to 796,825 (+)

**ROP23R1** *GCGGGTTTGAATGCAAGGTTTCGTGCTGATCAA*GCGGCCGC**CCAGCTCCGACTGTGAAGCC** Pru ROP23 KO 5’ target RP

**ROP23F2** *TTCTGGCAGGCTACAGTGACACCGCGGTGGAGG*GCGGCCGC**ACCGGAATCCGTGAAGACCTCC** Pru ROP23 KO 3’ target FP

**ROP23R2** *GTGAGCGGATAACAATTTCACACAGGAAACAGC*ACTAGT**CCTCAAGCCCACCAAACATTCCC** Pru ROP23 KO 3’ target RP

**ROP24F1** *TTGGGTAACGCCAGGGTTTTCCCAGTCACGACG*GTTTAAAC**CTGCATCATAGCCAACCATGCTGG** Pru ROP24 KO 5’ target FP pRS416.ROP24P TGME49_252360 chrIII 512,283 to 514,541 (+)

**ROP24R1** *GCGGGTTTGAATGCAAGGTTTCGTGCTGATCAA*GCGGCCGC**TGCCTCTCAAGGCATTGAGCGG** Pru ROP24 KO 5’ target RP

**ROP24F2** *TTCTGGCAGGCTACAGTGACACCGCGGTGGAGG*GCGGCCGC**CACTGACGGAACACCGTCCC** Pru ROP24 KO 3’ target FP

**ROP24R2** *GTGAGCGGATAACAATTTCACACAGGAAACAGC*ACTAGT**AAGCGTAGGATTCTCGCTGGGC** Pru ROP24 KO 3’ target RP

**ROP25F1** *TTGGGTAACGCCAGGGTTTTCCCAGTCACGACG*GTTTAAC**GGCTCGGAACACGCAGTAAGC** Pru ROP25 KO 5’ target FP pRS416.ROP25P TGME49_202780 chrVIIa 3,039,290 to 3,032,387(-)

**ROP25R1** *GCGGGTTTGAATGCAAGGTTTCGTGCTGATCAA*ACTAGT**GTACGGCGGTAGGACATTGACATC** Pru ROP25 KO 5’ target RP

**ROP25F2** *TTCTGGCAGGCTACAGTGACACCGCGGTGGAGG*ACTAGT**CCCAAGCTTCAGTGCTTCACGTTC** Pru ROP25 KO 3’ target FP

**ROP25R2** *GTGAGCGGATAACAATTTCACACAGGAAACAGC*GCGGCCGC**GCCAGCATGCGTGGAGTACAG** Pru ROP25 KO 3’ target RP

**ROP26F1** *TTGGGTAACGCCAGGGTTTTCCCAGTCACGACG*GTTTAAAC**GGCACCACGTGTAAGACCGC** Pru ROP26 KO 5’ target FP pRS416.ROP26P TGME49_211260 chrIV 1,886,258 to 1,889,338 (-)

**ROP26R1** *GCGGGTTTGAATGCAAGGTTTCGTGCTGATCAA*ACTAGT**AAGGGTTGCCCTACCGTGGTC** Pru ROP26 KO 5’ target RP

**ROP26F2** *TTCTGGCAGGCTACAGTGACACCGCGGTGGAGG*ACTAGT**GGTGACGTTGGGCTACCTTCTG** Pru ROP26 KO 3’ target FP

**ROP26R2** *GTGAGCGGATAACAATTTCACACAGGAAACAGC*GCGGCCGC**GGAACGATTGACCACGTCGCTC** Pru ROP26 KO 3’ target RP

**ROP27F1** *TTGGGTAACGCCAGGGTTTTCCCAGTCACGACG*GTTTAAAC**ACAACTGACACTCTCGGAAGC** Pru ROP27 KO 5’ target FP pRS416.GRA27P TGME49_313330 chrXI 3,273,286 to 3,280,282 (-)

**ROP27R1** *GCGGGTTTGAATGCAAGGTTTCGTGCTGATCAA*ACTAGT**TCACCTAGGCGTCAGGTCAGAC** Pru ROP27 KO 5’ target RP

**ROP27F2** *TTCTGGCAGGCTACAGTGACACCGCGGTGGAGG*ACTAGT**ACATAGAGACTCGGCCGACGC** Pru ROP27 KO 3’ target FP

**ROP27R2** *GTGAGCGGATAACAATTTCACACAGGAAACAGC*GCGGCCGC**TTGAGGGAGCCTCCTTGCTCC** Pru ROP27 KO 3’ target RP

**ROP28F1** *TTGGGTAACGCCAGGGTTTTCCCAGTCACGACG*GTTTAAAC**CTCGTGCACCGTTCACAATCCC** Pru ROP28 KO 5’ target FP pRS416.ROP28P TGME49_258370 chrVIIb 3,449,175 to 3,451,449 (-)

**ROP28R1** *GCGGGTTTGAATGCAAGGTTTCGTGCTGATCAA*GCGGCCGC**TCAGCAGACAGCCACCACTGG** Pru ROP28 KO 5’ target RP

**ROP28F2** *TTCTGGCAGGCTACAGTGACACCGCGGTGGAGG*GCGGCCGC**AGGATCGACGGTGGACACCG** Pru ROP28 KO 3’ target FP

**ROP28R2** *GTGAGCGGATAACAATTTCACACAGGAAACAGC*ACTAGT**TCACTCGGCTAGTGGCTATCTGTC** Pru ROP28 KO 3’ target RP

**ROP30F1** *TTGGGTAACGCCAGGGTTTTCCCAGTCACGACG*GTTTAAAC**GGCAGCGGACTTTAGTCCGG** Pru ROP30 KO 5’ target FP pRS416.ROP30P TGME49_227010 chrX 1,139,627 to 1,114,677 (+)

**ROP30R1** *GCGGGTTTGAATGCAAGGTTTCGTGCTGATCAA*GCGGCCGC**CTGCTGCGCTACCTCCTTCG** Pru ROP30 KO 5’ target RP

**ROP30F2** *TTCTGGCAGGCTACAGTGACACCGCGGTGGAGG*GCGGCCGC**GGCAGTTTTCATGGGCTCGAGG** Pru ROP30 KO 3’ target FP

**ROP30R2** *GTGAGCGGATAACAATTTCACACAGGAAACAGC*ACTAGT**GTAACGCTGTCATGCCTCGGC** Pru ROP30 KO 3’ target RP

**ROP31F1** *TTGGGTAACGCCAGGGTTTTCCCAGTCACGACG*GTTTAAAC**CCTCCAGCCATTACAGCAACAGC** Pru ROP31 KO 5’ target FP pRS416.ROP31P TGME49_258800 chrVIIb 3,177,592 to 3,178,594 (+)

**ROP31R1** *GCGGGTTTGAATGCAAGGTTTCGTGCTGATCAA*ACTAGT**CCCATGCTCAGTGTGTCGGAAC** Pru ROP31 KO 5’ target RP

**ROP31F2** *TTCTGGCAGGCTACAGTGACACCGCGGTGGAGG*ACTAGT**TTCCTGCAGGCATCCAGCAGC** Pru ROP31 KO 3’ target FP

**ROP31R2** *GTGAGCGGATAACAATTTCACACAGGAAACAGC*GCGGCCGC**TGGCCGCTCCTCCACTCTAG** Pru ROP31 KO 3’ target RP

**ROP32F1** *TTGGGTAACGCCAGGGTTTTCCCAGTCACGACG*GTTTAAAC**CCTGCGTCATGCGTACTACTGG** Pru ROP32 KO 5’ target FP pRS416.ROP32P TGME49_270920 chrVIII 4877333 to 4879945 (+)

**ROP32R1** *GCGGGTTTGAATGCAAGGTTTCGTGCTGATCAA*ACTAGT**CAGTGCCTCGCTATGCTGTCC** Pru ROP32 KO 5’ target RP

**ROP32F2** *TTCTGGCAGGCTACAGTGACACCGCGGTGGAGG*ACTAGT**GCCACAAGGTACATGCACTCACC** Pru ROP32 KO 5’ target FP

**ROP32R2** *GTGAGCGGATAACAATTTCACACAGGAAACAGC*GCGGCCGC**GAGTCACGGCAACACCAGCAG** Pru ROP32 KO 5’ target RP

**ROP35PF1** *TTGGGTAACGCCAGGGTTTTCCCAGTCACGACG*GTTTAAAC**AGTGACGCCCTCCGTTGACTG** Pru ROP35 KO 5’ target FP pRS416.ROP35P TGME49_304740 chrVIIa 678,437 to 681,309 (-)

**ROP35PR1** *GCGGGTTTGAATGCAAGGTTTCGTGCTGATCAA*ACTAGT**AAGCTCCCGTCAAGGACTGCAC** Pru ROP35 KO 5’ target RP

**ROP35PF2** *TTCTGGCAGGCTACAGTGACACCGCGGTGGAGG*ACTAGT**ACTTCTCGATCGACGATCCCGAC** Pru ROP35 KO 3’ target FP

**ROP35PR2** *GTGAGCGGATAACAATTTCACACAGGAAACAGC*GCGGCCGC**CGGTGGATTACGTTCGGTGCAC** Pru ROP35 KO 3’ target RP

**ROP36F1** *TTGGGTAACGCCAGGGTTTTCCCAGTCACGACG*GTTTAAAC**GCACTGTGCACCAGTGCTCG** Pru ROP36 KO 5’ target FP pRS416.ROP36P TGME49_207610 chrIb 151,792 to 153,817 (+)

**ROP36R1** *GCGGGTTTGAATGCAAGGTTTCGTGCTGATCAA*ACTAGT**CCTGTGCTGTCAACCGCCCTTC** Pru ROP36 KO 5’ target RP

**ROP36F2** *TTCTGGCAGGCTACAGTGACACCGCGGTGGAGG*ACTAGT**CGCCTATCGATGCTGTCAAGGG** Pru ROP36 KO 3’ target FP

**ROP36R2** *GTGAGCGGATAACAATTTCACACAGGAAACAGC*GCGGCCGC**GCAGAGTGGCGCAAGCTTCC** Pru ROP36 KO 3’ target RP

**ROP37F1** *TTGGGTAACGCCAGGGTTTTCCCAGTCACGACG*GTTTAAAC**CTTCGGTTGGGCAGCTACTGG** Pru ROP37 KO 5’ target FP pRS416.ROP37P TGME49_294560 chrIa 967,775 to 965,193(-)

**ROP37R1** *GCGGGTTTGAATGCAAGGTTTCGTGCTGATCAA*ACTAGT**GAGCTTGCTCCCGCTTGAATGG** Pru ROP37 KO 5’ target RP

**ROP37F2** *TTCTGGCAGGCTACAGTGACACCGCGGTGGAGG*ACTAGT**CGATGAACACCCCTTCTTTCAGGTG** Pru ROP37 KO 3’ target FP

**ROP37R2** *GTGAGCGGATAACAATTTCACACAGGAAACAGC*GCGGCCGC**GCGAGACCATGCTTACTGTCCC** Pru ROP37 KO 3’ target RP

**ROP38PF1** *TTGGGTAACGCCAGGGTTTTCCCAGTCACGACG*GTTTAAAC**GGTGGTCTGAGAGTTTGATCACGG** Pru ROP38 KO 5’ target FP pRS426.ROP38P TGME49_242110 chrVI 1,892,051 to 1,923,747(+) **ROP38PR1** *GCGGGTTTGAATGCAAGGTTTCGTGCTGATCAA*ACTAGT**AATTGTGGGGAGTCCGCTGGTG** Pru ROP38 KO 5’ target RP

**ROP38PF2** *TTCTGGCAGGCTACAGTGACACCGCGGTGGAGG*ACTAGT**TCGCTAACTGGGTAGCCTGCTG** Pru ROP38 KO 3’ target FP

**ROP38PR2** *GTGAGCGGATAACAATTTCACACAGGAAACAGC*GCGGCCGC**GCGACTGTCTTGCCTACCACTC** Pru ROP38 KO 3’ target RP

**ROP39F1** *TTGGGTAACGCCAGGGTTTTCCCAGTCACGACG*GTTTAAAC**GCCTGAGAATCCGGTCATGCTC** Pru ROP39 KO 5’ target FP pRS416.ROP39P TGME49_262050 chrVIIb 1,406,948 to 1,408,893 (+)

**ROP39R1** *GCGGGTTTGAATGCAAGGTTTCGTGCTGATCAA*ACTAGT**ACTGAACAGACGGCGGTCCAC** Pru ROP39 KO 5’ target RP

**ROP39F2** *TTCTGGCAGGCTACAGTGACACCGCGGTGGAGG*ACTAGT**GAGGCAAATCGGTCACCTCCAC** Pru ROP39 KO 3’ target FP

**ROP39R2** *GTGAGCGGATAACAATTTCACACAGGAAACAGC*GCGGCCGC**GAACTCACGGGCTCCAGGTG** Pru ROP39 KO 3’ target RP

**ROP40F1** *TTGGGTAACGCCAGGGTTTTCCCAGTCACGACG*GTTTAAAC**ATTCTGGGAGTTGGGCTACCGG** Pru ROP40 KO 5’ target FP pRS416.ROP40P TGME49_291960 chrIX 4,434,786 to 4,437,949 (+)

**ROP40R1** *GCGGGTTTGAATGCAAGGTTTCGTGCTGATCAA*ACTAGT**GTATCGTTTGCAGTGCCTGCAGC** Pru ROP40 KO 5’ target RP

**ROP40F2** *TTCTGGCAGGCTACAGTGACACCGCGGTGGAGG*ACTAGT**ACGGCTACCCTCCTCAGAACG** Pru ROP40 KO 3’ target FP

**ROP40R2** *GTGAGCGGATAACAATTTCACACAGGAAACAGC*GCGGCCGC**GAGATCCGTGTGCGACATCATGC** Pru ROP40 KO 3’ target RP

**ROP41F1** *TTGGGTAACGCCAGGGTTTTCCCAGTCACGACG*GCTAGC**GCGGCGTACATGCCACTTTGC** Pru ROP41 KO 5’ target FP pRS416.ROP41P TGME49_266100 chrIX 1,306,450 to 1,303,634 (-)

**ROP41R1** *GCGGGTTTGAATGCAAGGTTTCGTGCTGATCAA*GTTTAAAC**GACACATCAGTCGGAGCGGG** Pru ROP41 KO 5’ target RP

**ROP41F2** *TTCTGGCAGGCTACAGTGACACCGCGGTGGAGG*GTTTAAAC**GTGCACCCCGAAGTTCAGAGG** Pru ROP41 KO 3’ target FP

**ROP41R2** *GTGAGCGGATAACAATTTCACACAGGAAACAGC*GCGGCCGC**CACGAACCTGAATGCCGTCTGG** Pru ROP41 KO 3’ target RP

**ROP42F1** *TTGGGTAACGCCAGGGTTTTCCCAGTCACGACG*GCTAGC**ACGGCCGCGTAGTGGACAATG** Pru ROP42,43,44 KO 5’ target FP pRS416.ROP42P TGME49_209985 chr1b 1611696 to 1625260 (-)

**ROP42R1** *GCGGGTTTGAATGCAAGGTTTCGTGCTGATCAA*ACTAGT**TGATGAGGCGTTAGGCGGAACC** Pru ROP42,43,44 KO 5’ target RP

**ROP42F2** *TTCTGGCAGGCTACAGTGACACCGCGGTGGAGG*ACTAGT**CACGAGCCCGTGAGCAGGAAC** Pru ROP42,43,44 KO 3’ target FP

**ROP42R2** *GTGAGCGGATAACAATTTCACACAGGAAACAGC*TCTAGA**CAGAAGAGCGCTGGTCTGCGTA** Pru ROP42,43,44 KO 3’ target RP

**ROP45F1** *TTGGGTAACGCCAGGGTTTTCCCAGTCACGACG*GTTTAAAC**CGTCGTTGCGAGAAACAGTCCAG** Pru ROP45 KO 5’ target FP pRS416.ROP45P TGME49_281675 chrVIIa 4,250,829 to 4,257,240 (+)

**ROP45R1** *GCGGGTTTGAATGCAAGGTTTCGTGCTGATCAA*ACTAGT**CACCGCGATGCTGCTAGAGAC** Pru ROP45 KO 5’ target RP

**ROP45F2** *TTCTGGCAGGCTACAGTGACACCGCGGTGGAGG*ACTAGT**GAAACGCATCGAGCCCTTCCG** Pru ROP45 KO 3’ target FP

**ROP45R2** *GTGAGCGGATAACAATTTCACACAGGAAACAGC*GCGGCCGC**CGATGCCGCATGATCTCACACG** Pru ROP45 KO 3’ target RP

*FP indicates forward primer and RP indicates reverse primer. Italicized nucleotides indicate regions of crossover in yeast recombination cloning, underlined nucleotides indicate restriction enzyme sites, and bold nucleotides indicate specific priming target regions from the *Toxoplasma gondii* database (ToxoDB, version 26.0).
