# Supplementary material for: The Toxoplasma gondii Rhoptry Kinome Is Essential for Chronic Infection
Source: mBio. 2016 May 10;7(3):e00193-16. doi: 10.1128/mBio.00193-16 (PMC4959664; doi:10.1128/mBio.00193-16)
Supplement: Table S3 — Primers used to validate ROPK knockouts. The sequences of oligonucleotide primers used for validation of Δrop2/8, Δrop5, Δrop11, Δrop16, Δrop17, Δrop18, Δrop20, Δrop21, Δrop22, Δrop23, Δrop24, Δrop25, Δrop26, Δrop27, Δrop28, Δrop30, Δrop31, Δrop32, Δrop35, Δrop36, Δrop37, Δrop38/29/19, Δrop39, Δrop40, Δrop41, Δrop42/43/44, and Δrop45 knockouts are shown. The primers were designed from TgME49 data in toxodb.org. [file mbo002162811st3.docx]

**Primer Sequence Primer use Primer Sequence**

**Table S3. Primers used to validate *ROPK* knockouts.**

**Δrop26, Δrop27, **

**5’DHFRCXR**  **ACTGCGAACAGCAGCAAGATCG 5' RP for validation of all GOI 5' flank integrations**

**3’DHFRCXF** **GTTGGCCTACGTGACTTGCTGATG 3' FP for validation of all GOI 3' flank integrations**

**ROP2/8CXF CAGGGGATCAGATTCGGCGAC FP for validation of GOI 5' flank integration ROP28CXF CTGTGGTACACATGAGACTCGTGC**

**ROP2/8DF CTCCTGAGTACGAGCAACTGCG FP for GOI deletion validation ROP28DF GTGAAGGCAGGTCAAGCACCTAC**

**ROP2/8DR TCTCAATGGTGTCGCCTGACACG RP for GOI deletion validation ROP28DR GGATCGACTGGGTTTCATCCACTG**

**ROP2/8CXR** **CACCGGGTCACGCAATCGTC RP for validation of GOI 3' flank integration ROP28CXR CGCGTGCGTTGATGTAGAGTGC**

**ROP5CXF**  **GAATGAGAGCACGCGAAGCTGC ROP30CXF TCAGGCAGTGCTTCTAGTGTCAGTG**

**ROP5DF**  **CCTGCAGCGATGGTTGAGGC ROP30DF GCCGATGATGCTTGGCGACTTG**

**ROP5DR**  **TGTCCTGGACTCAGCTGAGCG ROP30DR GTAGTAGCAAACGATGCCCGCTG**

**ROP5CXR** **GCGAACGCGTACTTGTACGCTG ROP30CXR CGTCGATCATGACGCCATCATCC**

**ROP11CXF**  **GTGGCAGTGTAGAGCCATCGAC ROP31CXF CGTCAGTCTACAAGCAAGTCTGAGCA**

**ROP11PDF**  **ACTGGCTGCGCTAGGTCAGG ROP31DF TACTGTGGCATGCTTCGTCTGACG**

**ROP11DR**  **CTGACCCATCCATTGCCAGCAC ROP31DR GCCAGCTGACAGGACTGATGC**

**ROP11CXR** **TGTCCCGCATGTCGGTACCG ROP31CXR TCTCCTAGGCACAGGTGTCAGAC**

**ROP16CXF**  **GCAACTACTTCCGACGGAACCGTC ROP32CXF ACCATGGGTGCCACATCACTAGTC**

**ROP16PDF**  **CCCTGTTCAGAGCGCTACATTGG ROP32DF ACCAGGTGGCCACCATAGAACG**

**ROP16PDR**  **GGGAAACACTTCGTCAACAGCTGACTC ROP32DR AGGCATTCCGCGATGTGACCTAC**

**ROP16CXR** **CCTTAACAGGCAAATGAACACGAGCT ROP32CXR TGTCGAGACTGAACCCAACTCCTG**

**ROP17CXF**  **GCTGGAATTGACGCATCGCTCG ROP35CXF ACAGAGTCGATGTTCTAGACCGAGTG**

**ROP17DF**  **TGCGTTCGTGGTGAGCTAGGAC ROP35DF CTTACGTTGCCTCCGGTGAGC**

**ROP17DR**  **GACAGCGTATGGCATCTATGCCAC ROP35DR ACCCACTGCGGATCTCGTCC**

**ROP17CXR**  **CAGTGAGATGGCAGGTTGCCAG ROP35CXR CTGGTAAACGGCCTCGGTTCTC**

**ROP18CXF2**  **TGGCTGTGGTGAGGCACTCG ROP36CXF CACGTACTGTAGTCGTCCATCGC**

**ROP18DF2 GAGCGACAGAAAGCACTCGAGAC ROP36DF gtcaccgcagtccacgtatgac**

**ROP18DR2 GGAATTCTGGATGCGTCATCGGCA ROP36DR CCCAGGCACGTTTCCAGTGC**

**ROP18CXR2 TGGTTCCGGATACTGAGAGACACTAG ROP36CXR GTGCCTGCCGATAACAGGGTC**

**ROP20CXF ACAAATCTAGCCCGACCTATGATGCC ROP37CXF GATGTTGTGTCGCCTACCACAGG**

**ROP20DF** **CTGAGGGACACGCAGCTCAAG ROP37DF CTTCTGCGTCGAGCGCACAG**

**ROP20DR** **TCGCATGCCATAAGCGGCATTATC ROP37DR CGCTCTGAGTAGGATGGAAGCTCA**

**ROP20CXR** **CCTGTGTGTCAGTCGGATTAGC ROP37CXR AGCCTCGTTACTGCTGTGGTCG**

**Primer Sequence Primer use Primer Sequence**

**ROP21CXF ACCGGAGAATACGCTCTCCGC FP for validation of GOI 5' flank integration ROP38CXF GGTGAGCGCTATGGCGTTGC**

**ROP21DF** **CCAGCGTTGACAGCATCCAGC FP for GOI deletion validation ROP38DF CGTCAGGCAACGTGGTTGTACG**

**ROP21DR** **GCTCACCTTCTCGGCAGTCCA RP for GOI deletion validation ROP38DR ATGTTGCGGGTCCCTGTAGCG**

**ROP21CXR** **AGCGATTCCTCTCTGTATGTGCTCC RP for validation of GOI 3' flank integration ROP38CXR GCGCTCTTGCCGAAGTGTCG**

**ROP22CXF GGCGCATGTCGTTTACGTACTACTC ROP39CXF GCTAACCAACGGAAAGCACCCG**

**ROP22DF**  **CAGACCACTCGTTCGCCCAG ROP39DF CGCGAGCCACAAATGGTATCCC**

**ROP22DR**  **GCCAGGAAACGCGAACTTCTCAG ROP39DR GCGAAGAGATCACACAGCATCGG**

**ROP22CXR**  **GCTTCTATTGGAGGCGAAGACGTC ROP39CXR GGTTCAGTACCTCTGCAACTGGC**

**ROP23CXF CCATTCACGTGCACTCCATCCTG ROP40CXF CGGTGTATGCCGTCGCATTCC**

**ROP23DF**  **GATTCCAGGCGTCGTGTAATGCT ROP40DF CAGACACCAGCGTTAACTGACGAG**

**ROP23DR**  **CGAATACGCCTCGCTTGGGTTC ROP40DR TCAGCGACTTGACCGAGCGTC**

**ROP23CXR**  **TGAGCTAGACAGGTTCGACGTCC ROP40CXR AGCCGCTCTACAGTATCTCGTCC**

**ROP24CXF TGCTAGAAGTACGCTACACAGCGTC ROP41CXF GCTGATGTCATGGGTGGTTCGC**

**ROP24DF**  **CCTCGACTGCACATGACCGCTC ROP41DF GCTCTTGCTGTAAGCAAACGCATGG**

**ROP24DR**  **CATCAGCCCGAACGGTAGCAG ROP41DR CGATTCCGACGTCTGAAGGATCTG**

**ROP24CXR**  **ACGTCAATAGGTCGAAGGGACTGAG ROP41CXR CAGGTTGCTGTCGTAACTTGACTGC**

**ROP25CXF TCCTGTTTGCTCATCACAGTCGTG ROP424344CXF ATGACGATGCATGACCTTCCAAGACA**

**ROP25DF**  **CAAAAGGCGACGGCCAGTTCC ROP424344DF CGGGAGTCACTGACCGTGTTAGA**

**ROP25DR**  **AGTCCTTCTCCGTGCTGGTCG ROP424344DR AGTGGTGCTCTGAGAGATACCGTC**

**ROP25CXR**  **GCATTAGCACCCAGAACCGCC ROP424344CXR AGGCCGAACAGCCAGTGTTCTG**

**ROP26CXF ACCTGTATCTCACGTAAGCGGTAGAC ROP45CXF CGTCTTGTGTCTGCAGAGTCTCG**

**ROP26DF CAACTTCAGAGGTGTGCCACTCAG**  **ROP45DF CTGACAATTCGCGGACTCGGC**

**ROP26DR**  **GCACGGTCGCATTGAGCTGC ROP45DR GTTGCTGCAGACGAACCGGC**

**ROP26CXR**  **GACACAACTCAAGTGTCACCGGG ROP45CXR CGGTGCATGTTCCGATCGCAC**

**ROP27CXF CCAGCTCAGTATGTGTGCGACAG**

**ROP27DF**  **CGACAGAGACTTCCACCAAGCC**

**ROP27DR**  **TCCTTGCCCGTCAGAGAGGAAG**

**ROP27CXR**  **GACAGTCCGGAGCAAAGCCG**

*FP indicates forward primer and RP indicates reverse primer. ROP(GOI)CXF is the FP for validation of GOI 5' flank integrations, ROP(GOI)DF is the FP for GOI deletion validations, ROP(GOI)DR is the RP for GOI deletion validations, ROP(GOI)CXR is the RP for validation of GOI 3' flank integrations.
