# Supplementary material for: The Toxoplasma gondii Rhoptry Kinome Is Essential for Chronic Infection
Source: mBio. 2016 May 10;7(3):e00193-16. doi: 10.1128/mBio.00193-16 (PMC4959664; doi:10.1128/mBio.00193-16)
Supplement: Table S4 — Primers used to develop targeting plasmids for complementation. The sequences of oligonucleotide forward primers (FP) and reverse primers (RP) used for construction of Pru complementation vectors pURO5A (::ROP5A), pURO5C (::ROP5C), pURO17FLHA (::ROP17FLHA), pURO18 (::ROP18), pURO18KD (::ROP18KD), and pURO18RAH2(ATF) [::ROP18RAH2(ATF)] are shown. The corresponding TgME49 gene locus, chromosome, and nucleotides deleted for each knockout were determined from data in toxodb.org. [file mbo002162811st4.docx]

**Table S4. Primers used to develop targeting plasmids for complementation.**

**Primer Sequence Primer Use KO Construct TGME49 Replacement Locus**

**UPRPFF1** *TTGGGTAACGCCAGGGTTTTCCCAGTCACGACG*GTTTAAAC**GTGAGCTCATGCTGGAGCTTCG** Pru UPRT KO 5’ utr target FP all pRS416.URO 5' flanks TGME49_312480 chrXI 2,718,675 to 2,722,697 (+)

all pRS426.URO 5' flanks

**UPRPFR2** *GTGAGCGGATAACAATTTCACACAGGAAACAGC*GCGGCCGC**CTGGCGTTCGATCGACCGAAG** Pru UPRT KO 3’ utr target RP all pRS416.URO 3' flanks

all pRS426.URO 5' flanks

**UPRPFR1 AGCTTTCCGCTCGCTGGGAC** Pru UPRT KO 5’ target RP all pRS416.URO 5' flanks

**UPRPFF2** **GCCCTGCTGTCTTGTCAGGTACT** Pru UPRT KO 3’ target FP all pRS416.URO 3' flanks

**UROP17FX** *CGTATTCCTTTTTTCGTCGGACCTGTCCACAGGGCTTCTAAA***GTCCGAACGAAGCCACTAGTGACG** Pru ROP17 coding+utr FP pRS416.URO17FLHA TGME49_ 258580 chrVIIb 3,322,127 to 3,325,095 (-)

**UROP17RX** *CGCGATTCCGTCAGCGGTCTGTCAAAAAAACTAGAGACC*CCTAGGCTAAGCAGCAGCGTAATCTG

GAACATCGTATGGGTAGAGCTTGACTTTGTCATCGTCGTCCTTGTAGTCCCCGGG**CTCCTTCTGTA**

**ATAAAGCCGCCT** Pru ROP17 coding+FLHA tag RP

**URO18PXF1** *CGTATTCCTTTTTTCGTCGGACCTGTCCACAGGGCTTCTAAA***CGCGGAAGTAACTCGAGTCGATG** Pru ROP18 coding+utr FP pRS416.URO18 TGME49_205250 chrVIIa 1,516,845 to 1,514,369 (-)

**UROP18PXR1** *CGCGATTCCGTCAGCGGTCTGTCAAAAAAACTAGAGACC***GGCAATAATGCCATCCGTCGAGAG** Pru ROP18 codiing RP

**ROP18KDR TTCGCCGGTTTGATAGCCGTATGCACAATTCCCTGAGC** Pru ROP18 KD coding 5' frag 1 RP pRS416.URO18KD TGME49_205250 chrVIIa 1,516,845 to 1,514,369 (-)

**ROP18KDF GCTCAGGGAATTGTGCATACGGCTATCAAACCGGCGAA** Pru ROP18 KD coding 3' frag 2 FP

**ROP182NR TTGAGAAAAGTACCTCGGGATTCCGGACAGACGGGGTCTCGAGGAGCTA** Pru ROP18ΔATF2 coding 5' frag 1 RP pRS416.URO18ΔATF TGME49_205250 chrVIIa 1,516,845 to 1,514,369 (-)

**ROP182NF TAGCTCCTCGAGACCCCGTCTGTCCGGAATCCCGAGGTACTTTTCTCAAG** Pru ROP18ΔATF2 coding 3' frag 2 FP

**UPRT ROP5AR1** *CTAAAAACAAGTACATGGAAAGCGTGAATCGGTG*ACTAGT**AGCTTTCCGCTCGCTGGGAC** Pru 5'ΔUPRT::ROP5C target RP pRS426.UROP5A TGME49_308090 chrXII

**UPRT ROP5AF2** *TGCTGCGCCCTCAGTCGCTTACCCATACGATGTTCCAGATTACGCTTGA*ACTAGT**GCCCT**

**GCTGTCTTGTCAGGTACT**  Pru 3'ΔUPRT::ROP5C+HA target FP

**ROP5A CRF1** **CACCGATTCACGCTTTCCATGTACTTG** Pru ROP5 coding+utr FP pRS426.UROP5A TGME49_308090 chrXII

**ROP5A CRR1** *TCAAGCGTAATCTGGAACATCGTATGGGTA***AGCGACTGAGGGCGCAGCA** Pru ROP5 coding+HA RP

**UPRT ROP5CR1** *GTGGCTAGTCTAGCGAGCTTCGTCGCCATCTGGC*ACTAGT**AGCTTTCCGCTCGCTGGGAC** Pru ΔUPRT::ROP5C 5' target RP pRS426.UROP5C TGME49_308090 chrXII

**UPRT ROP5CF2** *CTCAGTCGCTTACCCATACGATGTTCCAGATTACGCTTGAGAAAACGGCTC*ACTAGT**GCCCT**

**GCTGTCTTGTCAGGTACT** Pru ΔUPRT::ROP5C 3'+HA target FP

**ROP5C CRF1** **GCCAGATGGCGACGAAGCTC** Pru ROP5 coding+5'utr FP pRS426.UROP5C TGME49_308090 chrXII

**ROP5C CRR1** *GAGCCGTTTTCTCAAGCGTAATCTGGAACATCGTATGGGTA***AGCGACTGAGGGCGCAGCA** Pru ROP5 coding+HA RP

*Italicised nucleotides indicate regions of crossover in yeast recombination cloning, underlined nucleotides indicate restriction enzyme sites, and bold nucleotides indicate specific genomic priming target regions corresponding to type II ME49 in the *Toxoplasma gondii* database (ToxoDB version 26.0). Corresponding replacement loci could not be pulled out for ROP5A and ROP5C since ME49 database is incomplete at this locus.
