# Supplementary material for: The Toxoplasma gondii Rhoptry Kinome Is Essential for Chronic Infection
Source: mBio. 2016 May 10;7(3):e00193-16. doi: 10.1128/mBio.00193-16 (PMC4959664; doi:10.1128/mBio.00193-16)
Supplement: Table S5 — Primers used to validate complementation of ROPK knockouts. The sequences of oligonucleotide primers used to validate integration of complementation vectors pURO5A (::ROP5A), pURO5C (::ROP5C), pURO17FLHA (::ROP17FLHA), pURO18 (::ROP18), pURO18KD (::ROP18KD), and pURO18RAH2(ATF) [::ROP18RAH2(ATF)] are shown. Primers were designed from TgME49 data in toxodb.org. [file mbo002162811st5.docx]

**Table S5. Primers used to validate complementation of *ROPK* knockouts.**

**Primer Sequence Primer use**

**UPRTDF1 TGACGTCGGGTGCCTACGTTC FP for UPRT deletion validation**

**UPRTDR1 CGACAGCTGCACTCGAAGACAC RP for UPRT deletion validation**

**UPRTCXF TCTCTCCCTGAGCTGCACGTG 5' FP for validation of all 5' flank integrations of ΔGOI Complementation Constructs at UPRT**

**UPRTCXR CCAGGTTCGACACTGGTCAGATG 3' RP for validation of all 3' flank integrations of ΔGOI Complementation Constructs at UPRT**

**RP17SEQR4 CATCACCGTCTGGTCACACAAGG 5' RP for validation of 5' flank integrations of Δ Complementation Construct**

**RP17SEQF4 GACTTCGCCAGTTGCCACGAC 3' FP for validation of 3' flank integrations of Δ Complementation Construct**

**RP18COMPXRP GAGGCATTTGACTGCAGTGTCTCG 5' RP for validation of 5' flank integrations of Δ8 Complementation Constructs**

**RP18COMPXF ACGCCTGAGCTGGTTCAAGACC 3' FP for validation of 3' flank integrations of Δ8 Complementation Constructs**

**RP5ASEQR1 CAAGTACATGGAAAGCGTGAATCGGTG 5' RP for validation of 5' flank integrations of Δ5A Complementation Construct**

**RP5ASEQF5 TGCTGCGCCCTCAGTCGCT 3' FP for validation of 3' flank integrations of Δ5A Complementation Construct**

**RP5CSEQR1 GAGCTTCGTCGCCATCTGGC 5' RP for validation of 5' flank integrations of Δ5C Complementation Construct**

**RP5CSEQF5 TGCTGCGCCCTCAGTCGCT 3' FP for validation of 3' flank integrations of Δ5C Complementation Construct**

*FP indicates forward primer and RP indicates reverse primer.
